# Lipid Clustering Correlates with Membrane Curvature as Revealed by Molecular Simulations of Complex Lipid Bilayers

**DOI:** 10.1371/journal.pcbi.1003911

**Published:** 2014-10-23

**Authors:** Heidi Koldsø, David Shorthouse, Jean Hélie, Mark S. P. Sansom

**Affiliations:** Department of Biochemistry, University of Oxford, Oxford, United Kingdom; McMaster University, Canada

## Abstract

Cell membranes are complex multicomponent systems, which are highly heterogeneous in the lipid distribution and composition. To date, most molecular simulations have focussed on relatively simple lipid compositions, helping to inform our understanding of *in vitro* experimental studies. Here we describe on simulations of complex asymmetric plasma membrane model, which contains seven different lipids species including the glycolipid GM3 in the outer leaflet and the anionic lipid, phosphatidylinositol 4,5-bisphophate (PIP_2_), in the inner leaflet. Plasma membrane models consisting of 1500 lipids and resembling the *in vivo* composition were constructed and simulations were run for 5 µs. In these simulations the most striking feature was the formation of nano-clusters of GM3 within the outer leaflet. In simulations of protein interactions within a plasma membrane model, GM3, PIP_2_, and cholesterol all formed favorable interactions with the model α-helical protein. A larger scale simulation of a model plasma membrane containing 6000 lipid molecules revealed correlations between curvature of the bilayer surface and clustering of lipid molecules. In particular, the concave (when viewed from the extracellular side) regions of the bilayer surface were locally enriched in GM3. In summary, these simulations explore the nanoscale dynamics of model bilayers which mimic the *in vivo* lipid composition of mammalian plasma membranes, revealing emergent nanoscale membrane organization which may be coupled both to fluctuations in local membrane geometry and to interactions with proteins.

## Author Summary

Cell membranes play important roles *in vivo* both in shielding the cell interior from the surrounding environment and in cell function through lipid components of the membrane having roles in controlling protein function, cell signaling etc. We employ molecular dynamics simulations to explore the behavior of biologically realistic membrane models. Our simulations reveal nano-domain clustering of the glycolipid GM3 and to a lesser extent of the anionic lipid phosphatidylinositol 4,5-bisphophate (PIP_2_). When including transmembrane proteins we are able to observe preferential interactions of known regulatory lipids (e.g. GM3, PIP_2_ and cholesterol) with the proteins. Membrane curvature is shown to be coupled to the local lipid composition, suggestive of a link between lipid nano-domains and membrane geometry.

## Introduction

Our growing knowledge of lipid-lipid interactions [1], [2] and of lipid involvement in the control of membrane protein function [3]–[8] highlights the importance of the complexity of composition, structure and dynamics of cell membranes. The large number of different lipid species *in vivo* has led to an understanding of the cell membrane as a multicomponent system, which is highly heterogeneous in the lipid distribution and composition [9]–[12]. Studies of lipids influencing protein function have revealed that the lipid components of cell membranes play key functional roles in cells [3]–[8]. The lipid composition of membranes differs between species, and also between the plasma membrane of mammalian cells and intracellular membranes such as those of the endoplasmic reticulum, nucleus, and mitochondria [9], [10]. This spatial dependency of membrane lipid composition further highlights its complexity and potential important role in cell function. It is also related to the observed spatial complexities of distribution of proteins within living cell membranes [13], [14].

The lipid compositions of the extracellular and the intracellular leaflets of plasma membranes are highly asymmetric [9]–[16]. Mammalian plasma membranes are composed of approximately 65% glycerolipids, 10% sphingolipids and 25% sterols such as cholesterol (Chol) [10]. The extracellular leaflet is enriched in phosphatidylcholine (PC) such as 1-palmitoyl-2-oleoyl-*sn*-glycero-3-phosphocholine (POPC) and 1,2-dioleoyl-*sn*-glycero-3-phosphocholine (DOPC), and in sphingolipids such as sphingomyelin (Sph) and glycosphingolipids. In contrast, the intracellular leaflet is enriched in phosphatidylethanolamine (PE) such as 1-palmitoyl-2-oleoyl-*sn*-glycero-3-phosphoethanolamine (POPE) and 1,2-dioleoyl-*sn*-glycero-3-phosphoethanolamine (DOPE), in phospatidylserine (PS) such as 1-palmitoyl-2-oleoyl-*sn*-glycero-3-phospho-L-serine (POPS) and 1,2-dioleoyl-*sn-*glycero-3-phospho-L-serine (DOPS), and in phosphatidylinositol (PI) including the di-phosphorylated lipid phosphatidylinositol-4,5-bisphosphate (PIP_2_). One consequence of this composition is that the inner leaflet of the plasma membrane is anionic in nature [7], [9], [10].

This compositional complexity is likely to result in a corresponding spatial and dynamic complexity, based on e.g. *in vitro* biophysical studies of lipid vesicles containing three or more lipid types [17], [18]. Molecular simulations provide a ‘computational microscope’ whereby the nanoscale details of the dynamic spatial distributions of lipids may be examined [19]. To date such simulations have largely focussed on relatively simple lipid compositions, thus informing our understanding of *in vitro* experimental studies [20]–[24]. In contrast, relatively few simulation studies have addressed the lipid asymmetry present *in vivo*, and these have generally focused on membranes containing only a few different lipid types [25]–[28]. Here we exploit a novel approach for modeling compositionally complex lipid bilayer membranes, which is fast, automated and allows for full control over lipid composition within both the outer and inner leaflets. This has enabled us to construct physiologically relevant membrane models which form the starting point for microsecond duration coarse grained (CG) molecular dynamics simulations [29]. In particular we focus on a complex asymmetric plasma membrane model, which contains the glycolipid GM3 (monosialodihexosylganglioside), within its outer leaflet. This plasma membrane model was simulated both alone and together with model proteins, revealing localised nano-domain formations of the GM3 within the outer leaflet and also of the key anionic lipid, phosphatidylinositol 4,5-bisphophate (PIP_2_), within the inner leaflet.

## Results

### A model plasma membrane

In order to explore the behavior of mixed lipid bilayers with a composition mimicking that of mammalian plasma membranes, a number of CG bilayer models containing 1500 lipid molecules were generated (see Supporting Information Table S1). These were derived from an initial model bilayer containing 1500 POPC molecules and with dimensions of ca. 20×20 nm which was generated via self-assembly simulations [30]. POPC molecules within either the upper or the lower leaflet were then randomly exchanged for other lipid species (see Methods for details). This yielded an asymmetric plasma membrane model composed of the lipid types abundant within the mammalian plasma membrane *in vivo*. Thus the overall lipid composition of the model plasma membrane (PM; Supporting Information Table S1) was POPC (25%), POPE (25%), POPS (7.5%), GM3 (5%), Sph (7.5%), Chol (25%) and PIP_2_ (5%). We also explored the effect of increasing the degree of lipid tail unsaturation by including DOPC, DOPE and DOPS lipids (PMUnsat; Supporting Information Table S1). The behavior of the asymmetric PM model has also been compared to that of symmetric lipid bilayer with compositions equivalent to either the upper (i.e. extracellular) or lower (intracellular) leaflets of PM in the PMUpper and PMLower simulations respectively.

**Figure 1:**
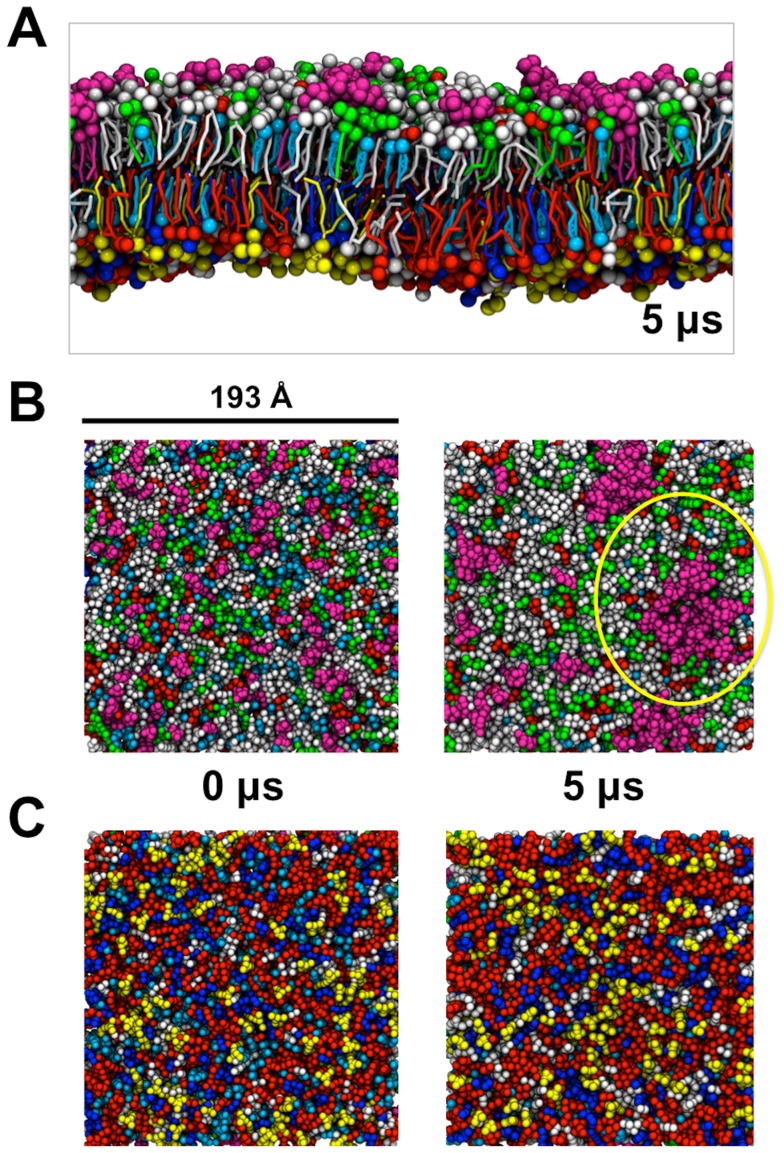
Initial and final structures of the plasma membrane. POPC is shown in light gray, POPE in red, Sph in green, GM3 in magenta, Chol in cyan, POPS in blue and PIP_2_ in yellow. (A) Side view of the plasma membrane at 5 µs. (B) View of the outer leaflet at 0 and 5 µs, with the GM3 cluster circled in the latter panel. (C) The inner leaflet at 0 and 5 µs.

Plasma membrane models consisting of 1500 lipids and resembling the *in vivo* composition were constructed and the CG simulations were run for 5 µs. (PM; Fig. 1A). The plasma membrane composition was POPC:POPE:Sph:GM3:Chol (40∶10∶15∶10∶25) within the outer leaflet and POPC:POPE:POPS:PIP_2_:Chol (10∶40∶15∶10∶25) within the inner leaflet. This membrane composition and distribution mimics a human plasma membrane (9). Two symmetric membrane models were additionally constructed and simulated as a reference for the asymmetric simulation; one system consisting of a symmetric bilayer with the same composition as the outer leaflet of the plasma membrane (PMUpper; Supporting Information Table S1) within both leaflets and one system with the same composition as the inner leaflet of the plasma membrane (PMLower; Supporting Information Table S1) within both leaflets.

### Cholesterol flip-flops

Cholesterol is able to flip-flop between the leaflets during the simulations. The cholesterol composition of both leaflets was the same in the initial setup. Even though many flip-flops occur during the simulation the overall percentage composition of the upper and lower leaflets remains almost the same throughout the simulations, with ∼49% in each leaflets and the remaining cholesterol located within the membrane core (see Supporting Information Table S2). The flip-flop rate for e.g. the 1500 lipid simulations is between 0.11–0.17 flip-flops/ns. The flip-flop rate in the PMProtein system is slightly slower than that observed for the protein-free membranes. As will be discussed later this is largely a result of favorable interactions between the protein and cholesterol within the bilayer. The initially equal distribution of cholesterol between leaflets remains the same during simulations of both the asymmetric and the symmetric lipid bilayers.

### Lipid nano-clusters

The change in the membrane organization within the asymmetric plasma membrane during the 5 µs simulation is shown in Fig. 1B–C. Most striking is the apparent formation of nano-domains of GM3 within the outer leaflet (Fig 1B). Large GM3 clusters up to 60 nm have also experimentally been proposed to occur in living cell membranes [31] and on the basis of X-ray scattering studies of GM3 bilayers this has been suggested to be due to strong and cooperative head group interactions [32]. In contrast, the inner leaflet lipids seem to retain more random distribution of the lipids. Similar patterns of behavior were observed within the symmetric membranes resembling the outer and inner leaflets (PMUpper & PMLower).

**Figure 2:**
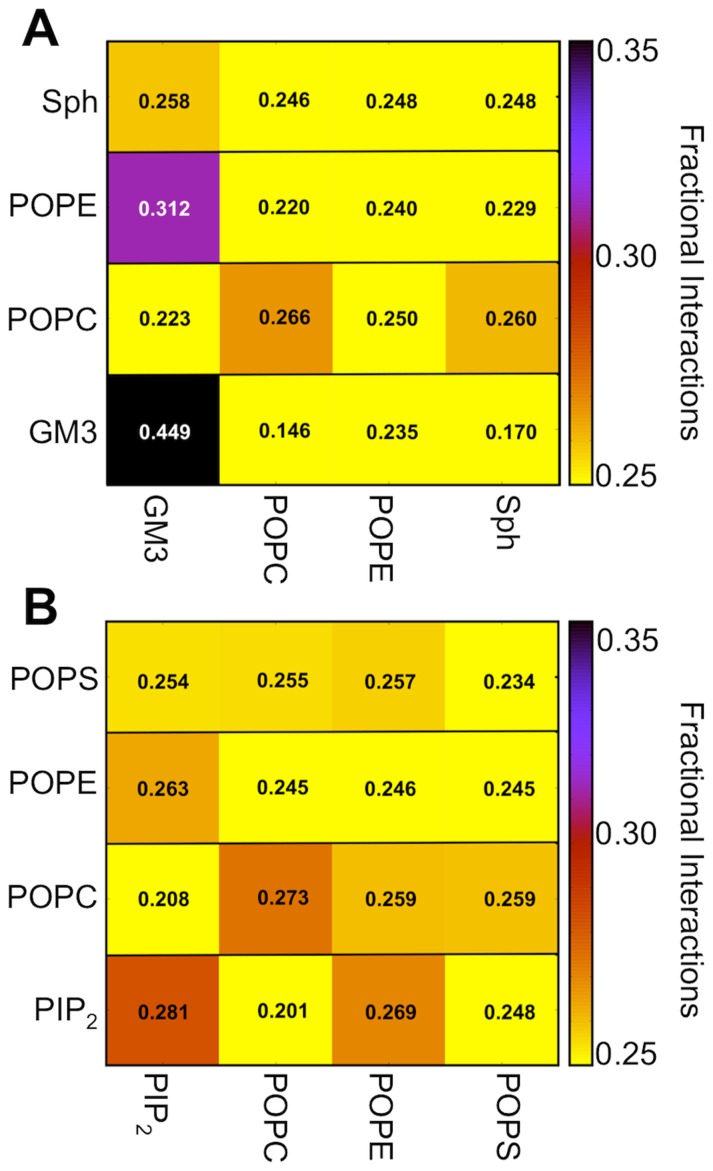
Fractional interaction matrix of the outer and inner leaflet of the plasma membrane. The matrix shows the fractional interaction as the relative number of contacts between lipids compared to all other contacts. If a lipid has more than one contact with another lipid this interaction is only counted once. Two lipids are defined as being in contact if the distance between the glycerol ester moiety and amino alcohols is less than 11 Å. Since cholesterol flip-flops between the leaflets during simulations it is not possible to assign these to specific leaflets and has therefore been omitted from this analysis. A fully random distribution of between four lipid types would result in a fraction of 0.25. (A) Fractional interaction of the lipids within the outer leaflet. (B) Fractional interaction of inner leaflet lipids.

This local clustering of GM3 is further supported by analysis of fractional interactions between different lipid types (Fig. 2A). Thus, approximately 45% of the lipid-lipid interactions of GM3 are with another GM3 molecule. All other lipid types within the outer leaflet are nearly randomly distributed, as indicated by approximately 25% fractional interaction with all other lipids. We did not observe any preferential Sph-Sph interactions within the simulations, even though this has previously been suggested [2], [33], [34]. This co-clustering of Sph is thought to be driven by the ability to form a hydrogen-bonding network through the hydroxyl group of the tails [34]. Our failure to observe this may therefore reflect a limitation of the current CG model for Sph. As mentioned above, cholesterol flip-flops during the simulation and was therefore not assigned to a unique leaflet and not included within the fractional interaction analysis.

The simulations of the PMUpper symmetric bilayer illustrate the same behavior with significant GM3 interactions while the other lipids were almost randomly distributed (Supporting Information Fig S1A, B). Similar analysis of the inner leaflet interactions revealed that the anionic PIP_2_ molecules form interaction networks with each other. However they do not form as large clusters as observed for GM3 Thus the fractional interaction between two PIP_2_ lipids is approximately 30% (Figure 2B). Interestingly, a similar degree of preferential interaction is observed for the POPC-POPC interactions within the inner leaflet in the asymmetric PM and in the symmetric PMLower membrane simulations (Fig. 2 and SI Fig. S1C, D) but is absent from the outer leaflet of PM and from PMUpper despite the higher content of POPC in the upper leaflet.

The distribution of the GM3 cluster sizes was assessed for the PM simulations. The clustering of GM3 was calculated over time utilizing a density-based clustering approach with a cutoff radius of 15 Å and a density requirement of 3 lipids (see Methods for details). The distribution of GM3 clusters over time is shown in the Supporting Information Figure S2A. There are a total of 75 GM3 lipids within the outer leaflet of the PM system. Based on the cluster distribution we see that the number of ‘free’ lipids (defined as a cluster size 1–3) equilibrates to a value of approximately 20% of the total lipids after 1 µs. This indicates that the remaining 80% of GM3 co-clusters into domains of 4–20 lipids, 21–40 lipids or bigger. This analysis also demonstrates that we observe convergence of the GM3 clustering i.e. the GM3 molecules are not evolving towards a single large cluster. As observed from the fractional interaction analysis, PIP_2_ molecules also cluster but into smaller domains, such that more than ∼50% of PIP_2_ molecules are distributed into clusters of between 4 and 20 lipids, which remain the same throughout the entire simulation (Supporting Information Fig S3A).

To evaluate the robustness of the clustering of GM3, the PM simulations were repeated with the terminal N-acetyl-neuraminic acid group of the headgroup in either a protonated (i.e. neutral) or a deprotonated (anionic) state (and with a minor difference in headgroup restraint parameters). Similar to what others have observed for simulations of cardiolipin-containing bilayers [35], we did not observe any significant differences in the behavior of the lipids within the membrane systems dependent on the presence vs. absence of the negative charge on the GM3 headgroup. When the GM3 headgroup is charged the clustering is accompanied by Na^+^ counter ions present in the system. Similar behavior is seen for PIP_2_. This reflects charge neutralization rather than specific lipid-ion interactions, as might be expected given the inherent approximations in the CG model of ions. This suggests that sodium ions and water may facilitate the stabilization of GM3 nano-domains. Indeed sodium ions and water are observed to form stabilizing interactions with the anionic head groups of GM3.

We also explored possible effects of lipid tail length and saturation by introducing lipids with di-unsaturated tails instead of just mono-unsaturated lipids. This yielded the PMUnsat model (see SI Table S1) with a composition of POPC:DOPC:POPE:DOPE:Sph:GM3:Chol (20∶20∶5∶5∶15∶10∶25) within the upper leaflet, and of POPC:DOPC:POPE:DOPE:POPS:DOPS:PIP_2_:Chol (5∶5∶20∶20∶8∶7∶10∶25) within the lower leaflet. The fractional interactions between the lipids in this simulation illustrate a similar behavior to that seen for the simpler PM simulation containing just mono-unsaturated lipids (Fig. 2 and SI Fig. S1E, F). Again, significant inter-GM3 interactions were observed within the outer leaflet alongside some degree of inter-PIP_2_ interactions within the inner leaflet. This suggests that the head group functionality is most likely the most dominating factor for the localized nano-domain interactions. No separation between the mono- and di-unsaturated lipids was observed in these simulations. This is not surprising since previous studies of symmetric lipid bilayer and monolayers have revealed that modification of lipid tail particle types is a necessity for phase separation of the lipids into raft-like domains [22], [24], [36], [37], indicating that the current CG model is not able to fully capture all such differences in lipid saturation.

### Protein interactions within a plasma membrane model

**Figure 3:**
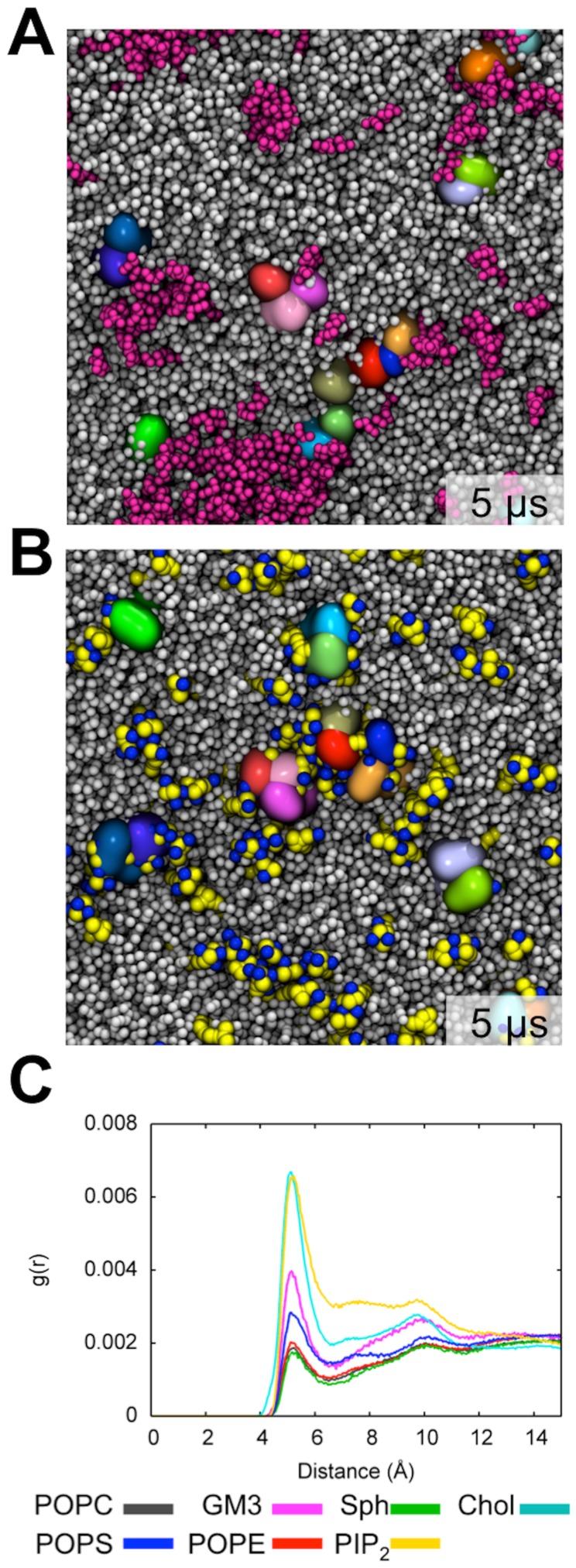
Lipid organization within a protein containing plasma membrane model after of 5 µs of simulation. (A+B) End state of simulations of a 1900 lipid plasma membrane containing sixteen membrane-spanning gp130 receptor proteins. The proteins are colored in different colors and represented by surfaces. The proteins were originally distributed on an evenly spaces grid with 60 Å between proteins. (A) Upper leaflet after 5 µs of simulations time. Proteins are shown in different colors and GM3 is shown in magenta spheres, while the rest of the lipids are shown in gray. (B) Inner leaflet after 5 µs of simulations. PIP_2_ is shown in yellow spheres and blue spheres illustrate sodium ions within 5 Å of PIP_2_. (C) Radial distribution functions of lipids around the protein.

To explore the possible influence of a simple transmembrane protein [38] on the properties of the model plasma membrane, a system containing sixteen single α-helical transmembrane domains (TMDs) was studied. The TMD chose was from a signaling protein, the cytokine receptor gp130. This was selected on the basis of extending earlier studies of anionic lipid clustering [38], and more importantly because signaling receptors (including e.g. receptor tyrosine kinases such as the EGFR), are thought to interact with a number of lipid types [6] and to co-localize with lipid nano-domains [39]. The TMDs were initially placed on a regular grid, with 60 Å between each protein to ensure no bias was introduced into the initial protein-protein interactions. This membrane system after 5 µs of simulations clearly illustrates that the proteins do *not* prevent GM3 cluster formations. Indeed, the proteins were observed to co-cluster together with GM3 (Fig 3A). The proteins associate into mainly dimers, and a single stable trimer throughout most of the simulation. The largest protein cluster observed being a trimer of dimers. The distribution and stability of protein cluster sizes can be seen in Supporting Information Fig. S4. The protein clusters were observed to be surrounded by GM3 molecules (Fig. 3A). Similarly to what we previously observed in a simpler system setup [38], the basic C-terminal of the gp130 TMD is able to attract anionic lipids, which can be illustrated by the interaction networks observed between protein and PIP_2_ within the inner leaflet of the plasma membrane (Fig. 3B). The PIP_2_ clusters do however not increase significantly in size as a result of the TMD proteins (Supporting Information Fig. S1J) and both the GM3 and PIP_2_ clusters converge to a similar size as seen in the PM system (Supporting Information Fig. S2B and S3B). GM3 and PIP_2_ have previously been described to be important in regulation of protein function [5], [6]. In addition to PIP_2_ and GM3, cholesterol is also observed to form favorable interactions with the protein as judged from radial distribution functions of the different lipid types around the protein (Fig. 3C).

**Figure 4:**
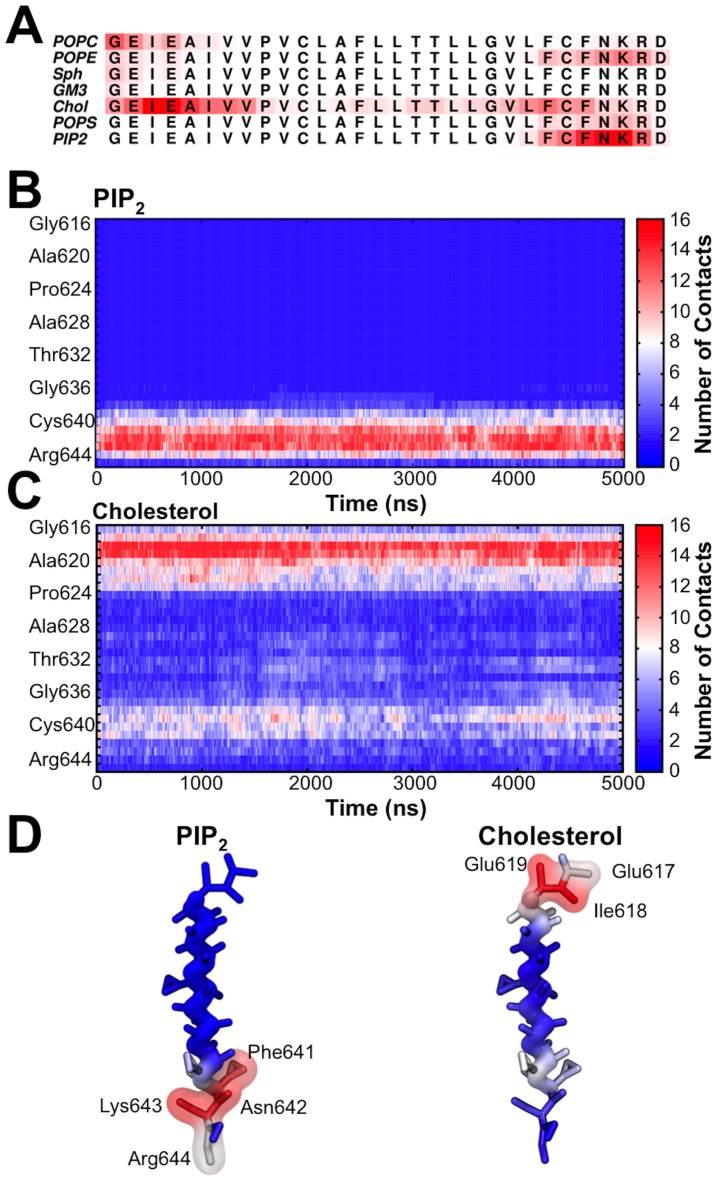
Protein-lipid interactions within a plasma membrane model. Interactions within 6 Å between protein and lipids head groups. (A) The average number of interactions between the protein and lipids mapped into the sequence. (B) Number of interactions between the PO3 bead of PIP_2_ and amino acid residues within the proteins. (C) Number of interactions between the ROH bead of cholesterol and the residues within the proteins. (D) The average number of contacts between the protein and PIP_2_ and cholesterol has been mapped into the protein structure. Interactions that are present in more than 50% of the entire simulations have been shown as surfaces.

The interaction between the lipids and proteins were further assessed by analyzing the average number of interactions between all proteins and the head group of the different lipid species. The average number of interactions of each lipid species has been mapped onto the amino acid sequence of the protein (Fig 4A). Similar to what was seen from the radial distribution function, it was observed that in particular cholesterol and PIP_2_ interacted strongly with the protein. As mentioned above, we noted that cholesterol is able to flip between the leaflets during the simulation [40] and consequently forms interactions along the TMD sequence within the membrane center. PIP_2_ form favorable interactions with the basic amino acid enriched C-terminus of the protein similar to previous observations for POPS in a simpler membrane composition [38]. Furthermore, POPC formed many interactions with the protein within the extracellular leaflet. This is nevertheless not surprising as POPC accounts for 40% of the lipids within the outer leaflet. From the radial distribution function analysis, GM3 appeared to interact relatively favorably with the protein. This is not mirrored in the interactions of the anionic head group of GM3 with the protein TMD. This suggests that GM3 interactions with the TMDs are mainly mediated via the lipid tails and/or at the interface between the tails and the head groups.

The interactions between cholesterol, PIP_2_ and the protein are illustrated in greater detail in Figure 4B–D. Favorable interactions between the PIP_2_ and protein molecules form quite quickly and remain stable during most of the simulations (Fig 4B). A similar pattern is observed from the cholesterol interactions, and as also seen in Fig. 4A, we observe some interactions of the head group of cholesterol within the membrane embedded parts of the protein as a result of the ability of cholesterol to flip into the membrane core. The average number of interactions has been mapped onto the protein structures in Fig. 4D, highlighting attraction of anionic lipids by the basic C-terminus of gp130 and strong interactions both within the N- and C-termini of the proteins with cholesterol.

### Lipid diffusion

It is evident from the results mentioned above that GM3, and to a lesser extent PIP_2_, form favorable interaction networks independently of membrane asymmetry, lipid tail saturation, bilayer size, and the presence or absence of proteins. It would be expected that the diffusion of lipid within nano-domains would be slower than freely moving lipids. To assess this, the mean square displacement (MSD) of the different lipids components was calculated during the 5 µs of simulations in the six different plasma membrane simulations (Supporting Information table S3). Within the plasma membrane the diffusion of GM3 (D = 1.5×10^−7^ cm^2^/s) is reduced by ∼40% compared to most other lipid types (D = 2.6×10^−7^ cm^2^/s). Also, PIP_2_ is observed to have a slightly slower diffusion (D = 2.0×10^−7^ cm^2^/s). The diffusion of the lipids we observe in the plasma membrane models is slower than that previously described in CG simulations of membranes. In simpler membrane models the diffusion of the lipids in pure membranes or with very few proteins is around 9−10×10^−7^ cm^2^/s [38] while it is decreased down to 4×10^−7^ cm^2^/s in highly protein crowded membranes [41]. The slower diffusion in complex membrane models, and especially for GM3, is most likely caused by the formation of lipid nano-domains and its larger head group able to interact with the surrounding water. The same approximately 40% reduced diffusion of GM3 relative to the other lipids is observed in all the simulations containing this glycolipid. One of the slowest diffusion constant of the anionic PIP_2_ lipid is observed in the simulations containing the membrane-spanning region of sixteen gp130 receptors (D = 1.5×10^−7^ cm^2^/s). Interactions between the basic C-terminal and anionic lipids were previously described in details [6], and this type of interaction is most likely the reason for the observed decrease in diffusion of PIP_2_. The single molecule diffusion constant of the glycolipid GM1 within live plasma membrane cells has been determined to 5×10^−9^ cm^2^/s [42]. The difference observed in our simulations and the experiments is likely to be due to both crowding effects of proteins and interactions with cytoskeletal proteins *in vivo*.

The evolution of the lipid diffusion was explored by calculating the diffusion coefficients for consecutive time intervals of 1 µs (Supporting Information Fig. S5). The diffusion of GM3 and PIP_2_ are slower than for the other lipids throughout all of the simulations. This suggests that both the more complex head groups (and hence more extensive interactions), the clustering of GM3 and PIP_2_ and in the case of PIP_2_ protein interactions lower their diffusion rates.

### A 6000 lipid plasma membrane model

**Figure 5:**
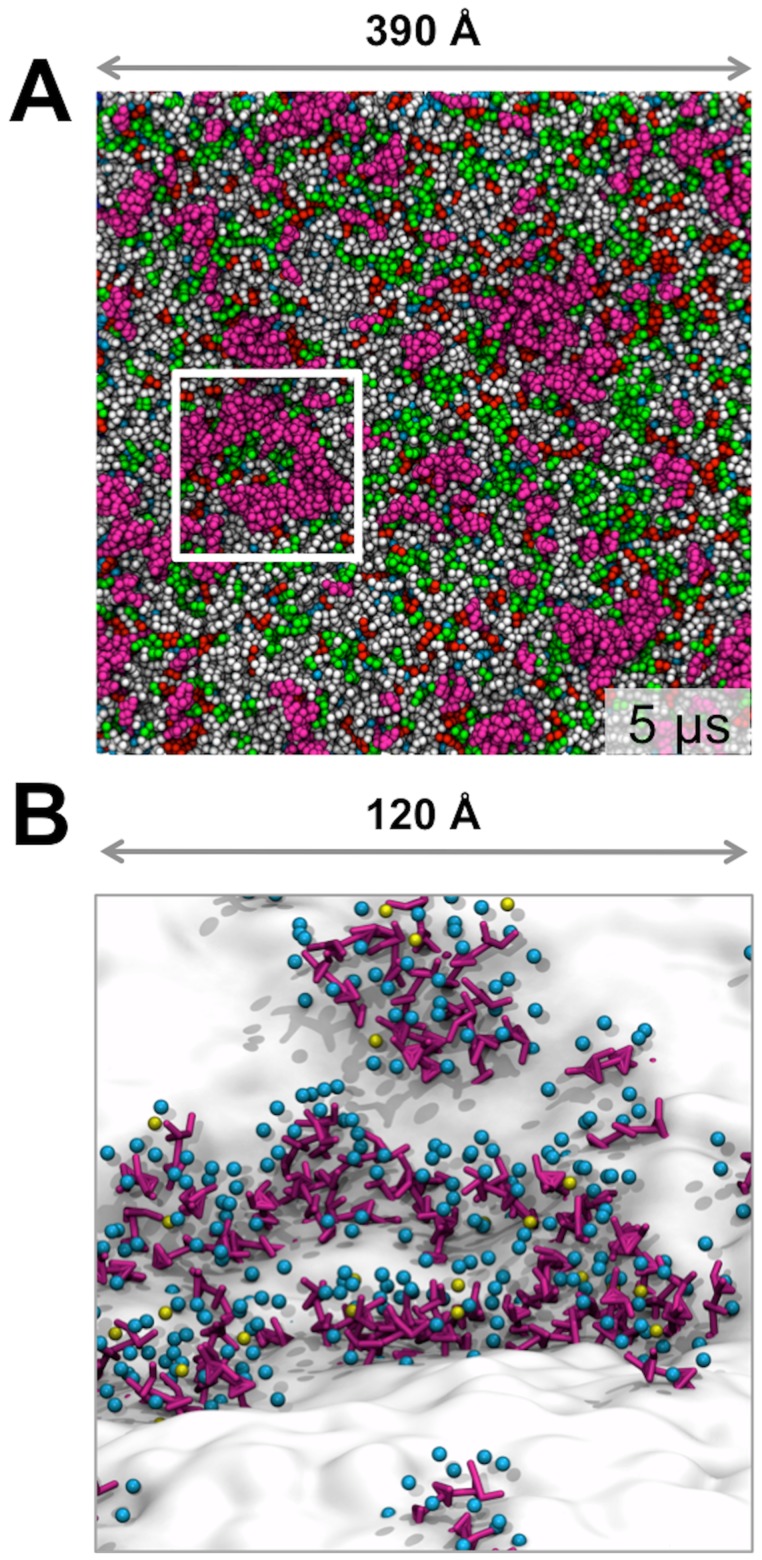
Lipid organization and interactions between GM3 head groups in a 6000 lipid plasma membrane model. (A) Plasma membrane composed of 6000 lipids. The same color scheme as Fig. 1 has been applied. (B) Zoom in (see white box of approximately 12 nm in (A)) on interactions between GM3 head groups within the lipid nano-domains. GM3s are represented as magenta coloured sticks. Water beads within 5 Å of GM3 have been shown in cyan and sodium ions within 5 Å of GM3 are shown in yellow. The entire membrane is shown as a white surface.

To better understand the effects of the size of the simulated bilayer patch on membrane behavior and lipid clustering, we performed a simulation of a substantially larger membrane patch consisting of 6000 lipids. This larger membrane system contained more than 150,000 particles with an area dimension of 39 nm ×39 nm and was run for 5 µs. The same overall behavior (including diffusion coefficients) was observed for this larger system compared to the 1500 lipid plasma membrane (Fig. 5). Inter-GM3 interactions were again shown to account for more than 40% of all interactions revealing similar clustering behavior as seen for the 1500 lipid simulations (Supporting Information Fig. S1G). Again large nano-domains of GM3 were observed. The interactions between the head groups are tightly mediated by water and sodium ions (Fig. 5B). As seen for the PM system, the number of *non-clustered* GM3 lipids converges to approximately 20% with the rest of the GM3 lipids participating in clusters (Supporting Information Fig. S2C).

This simulation allows us to investigate larger-scale emergent properties of the PM model. In our simulations of the PM6000 system we observe curvature of the membrane bilayer, as has been observed in other simulations of large (but simpler) lipid bilayers [43]–[45]. Local curvature of the membranes occurs within the first 0.1 µs of simulation time and continues to fluctuate dynamically over the course of the simulation. This curvature is unlikely to be a tension bias arising from the initial configuration of the complex membrane, as the area per lipid is expected to be the same for both the inner and outer leaflets with the lipid composition employed (see Methods and Table S1). Furthermore, the local curvature observed is irregular, rather than a global uniform deformation in one direction, again suggesting local dynamic fluctuations rather than an overall curvature bias in the system as a whole.

**Figure 6:**
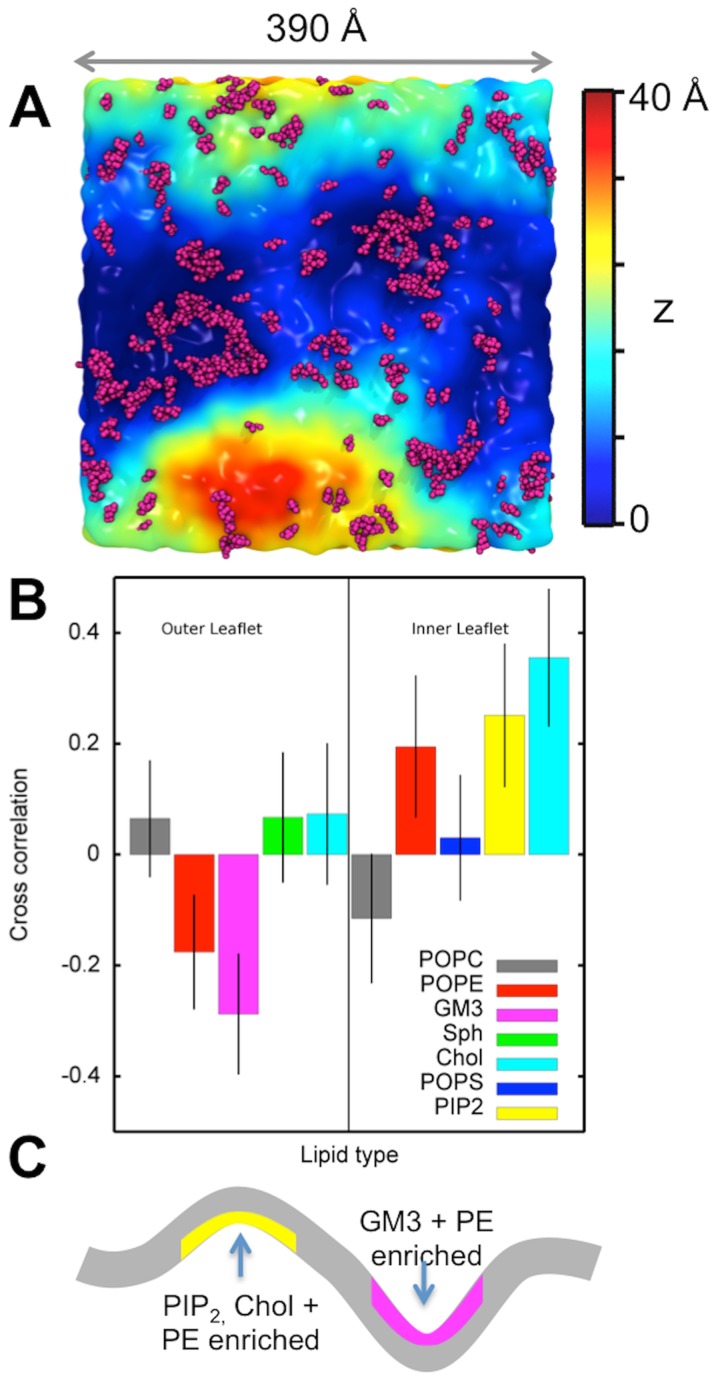
Membrane curvature and lipid distribution. (A) Top view of the outer leaflet of the PM6000 membrane colored according the z-position of the interface between the tails and head groups. GM3 is shown in magenta. (B) Cross-correlation between the z-coordinate and the lipid composition of the PM6000 simulation illustrated in (A). (C) Schematic illustration of the correlations between local bilayer geometry and local lipid composition. Thus the concave (downwards) deflections of the bilayer are locally enriched in GM3 and to a lesser extent PE in the outer leaflet of the bilayer, whilst the concave (upwards) deflections are enriched in PIP_2_, and PE in the inner leaflet of the bilayer.

Visualization of the simulation (Fig. 6A) suggested a correlation between the curvature in the PM6000 bilayer surface and the clustering of lipid molecules. Viewed from the extracellular (upper) surface, the bottom of the waves (i.e. the concave surfaces) were enriched in GM3 whilst if one views from the intracellular (lower) side the concave surfaces are enriched in PIP_2_ and cholesterol. We quantified this by calculations of the cross correlation between the local displacement of the membrane lipids from their average position along the bilayer normal (z) and the local composition of the bilayer (see Fig. 6B and Methods for details). This analysis revealed clear correlations between local bilayer geometry and local lipid composition. Thus the concave (downwards) deflections of the bilayer from the extracellular side were locally enriched in GM3 and to a lesser extent PE in the outer leaflet of the bilayer, whilst the concave (upwards) deflections from the intracellular side were enriched in PIP_2_, cholesterol and PE (Fig. 6C). Thus we would expect the GM3 and PIP_2_ clusters to be anti-correlated, which can be seen from the correlation matrix between the lipids within each leaflet (Supporting Information Fig. S6). The pattern of local enrichment of PE corresponds to the known preference of this lipid for an inverted hexagonal (H_II_) phase [46]. Furthermore, GM3 micro domains have been observed in mixed lipid bilayers by AFM imaging [47], and PIP_2_ has been shown to have direct effects on bilayer properties [48]. Cholesterol is believed to be involved in nano-domain formation [2], [33] and the clear correlation between curvature and composition observed may indicate that local cholesterol enrichment has an impact on the geometry of the membrane. The behavior of GM3 and PIP_2_ in our simulations is presumed to reflect the local clustering seen in the smaller scale simulations, mediated in part by Na^+^ and water (Fig 5B). However, note that we observe similar behavior in the simulations where GM3 is modeled as neutral and with slightly different head group parameters (data not shown). The local membrane curvature does not lead to local thinning or thickening of the bilayer.

## Discussion

In our simulations of complex plasma membrane-like lipid bilayers we observe asymmetric formation of lipid nano-clusters. The location of these nano-clusters within the bilayer correlates with local bilayer distortion/deflection. It seems that the formation of glycolipid nano-clusters in the outer leaflet is (largely) independent of the less pronounced clustering of PIP_2_ lipids within the inner leaflet mediated by sodium ions and water. Lipids within one leaflet are able to form local clusters independently of the composition of the opposite leaflet. Our simulations also illustrate this, since GM3 forms a tight network in both symmetric and asymmetric bilayers, and additionally in membranes containing protein. A similar type of glycolipid clustering (of GM1 [49]) was recently observed by others [24]. Similarly, PIP_2_ has been shown from experiments and simulations to cluster dependent on the presence of interacting membrane proteins [50]. Our results extend these previous studies in that we are for the first time able to study clustering of glycolipids and PIP_2_ simultaneously in a complex asymmetric membrane model. We are not only able to show local clustering as previously observed, but also the effect of membrane proteins and the anti-correlation between these types of nano-domains and their spatial organization with respect to membrane curvature.

Asymmetric membranes have been investigated in a number of previous computational studies [25]–[28], but not in bilayers of comparable complexity to those studied here. Through the simulations described here, we have been able to capture the clustering of GM3 comparable to that observed in cell membranes [31], although the cluster sizes of GM3 in our simulations are of the order of ca. 50–100 Å, compared to ca. 600 Å in the experimental studies. The differences may reflect the greater complexity of the cell membrane and its interactions but also the resolution limit of the experimental method applied.

When we introduce model proteins into the PM bilayer model we are able to see the co-clustering of proteins and lipids. We also see that the large number of different lipid types slows down the diffusion of the lipids compared to that in membranes of simpler lipid composition [38], [41], and that the local clustering of GM3 leaves this lipid less dynamic than other lipid types. Residues of the model TMD are both able to favorably cluster with glycolipids on the extracellular side of the membrane mainly through the tails in addition to forming favorable interactions with anionic lipids within the inner leaflet. Cholesterol is observed to form persistent interactions with the proteins on both the extracellular and the intracellular side. This suggests that there may be a cholesterol interaction site on the surface of the TMD of the signaling protein gp130. Of course, the limitations of the CG force field apply to cholesterol and its interactions (see e.g. [51]), so one might more cautiously conclude that the gp130 TMD exhibits a possible sterol interaction site, the specificity of which may be explored in more detail in future atomistic simulations. Thus the effects of local and asymmetric clustering of lipids on protein function may correlate both via specific protein-lipid interactions but maybe also because of the slower diffusion once captured in nano-domains. This is clearly related to on-going discussion of both direct (protein binding) and indirect (bilayer mediated) effects of PIP_2_ on membrane protein function [48] and organization [50].

The MARTINI force field has previously been shown to too ‘sticky’ for the interactions of globular proteins in aqueous solution [52]. However, simulated protein-lipid interactions [53], [54] and protein-protein interactions within the lipid bilayer [55]–[57] seem to reproduce experimental results quite well. We are therefore reasonably confident that the lipid-lipid clustering we observe within these simulations are not simply artefacts of the CG model. We observe that the clustering of GM3 is independent of lipid head group charge and is mediated by both water particles and sodium ions (Fig 5B). Similarly the clustering of PIP_2_ is bridged by sodium ions and sensitive to introduction of proteins within the system (Fig 3B). We therefore suggest that the lipid clustering observed in this study a result of favorable interaction between lipids of certain shape and charge, as has also been shown from experiments. The degree of clustering of GM3 that we observe seems to have converged in that that the number of unclustered lipids equilibrates towards 20% during the simulations. However, a limitation of the current CG representation is the difficulty in modelling phase separations when changing the tail saturation of the lipids. For example DOPC and DPPC phase separate *in vitro* but not in MARTINI. Additionally we are not able to capture the experimentally observed sphingomyelin co-clustering within our models. Both of these limitations indicate that important but subtle difference in lipid tails may not be sufficiently captured in CG simulations using the MARTINI and related force fields and hence the electrostatics, size and charge of the lipid head groups are main driving force in our observations of lipid clustering.

A further possible limitation is that of convergence of these complex bilayer simulations. A number of recent studies have discussed some of the difficulties of assessing convergence in complex membrane simulations (e.g. [58]): to some extent it is a question of assessing “unknown unknowns” [59]. However, as discussed above, the number of free (i.e. unclustered) lipid molecules plateaus with respect to time for both GM3 and PIP_2_. This suggests that the clustering we do observe is unlikely to be bias from the initial configuration of the simulation, but rather is a genuine local property of a complex bilayer, especially as our simulation systems do not seem to simply drift towards a single large cluster.

In summary, simulations now allow us to explore the nanoscale dynamics of model bilayers, which mimic the *in vivo* lipid composition of cell membranes. In these simulations we can see indications of emergent larger scale membrane organization which may be coupled both to fluctuations in local membrane geometry and to interactions with proteins. It will be of interest to extend these studies to higher levels of protein crowding [41] to better understand the interplay of compositional complexity and local spatial clustering of both lipids and proteins on aspects of membrane protein function.

## Methods

### Complex membrane generation

The complex membranes were generated by starting from a self-assembled POPC bilayer which was then ‘edited’. Thus, for lipids with the same or shorter chain length, the required coordinates were simply transferred from a randomly selected POPC molecule and the lipid was relabeled to e.g. POPE, POPS, PPCS, or POPE and the particle types altered accordingly. For larger lipids (e.g. PIP_2_, GM3, DOPC, DOPE, DOPS) the new lipid molecule was superimposed on the first two tail beads of the randomly selected POPC molecule and the two lipid molecules (new and POPC) were exchanged. Cholesterol was superimposed on the head group and 3 of the hydrophobic tail beads of a randomly selected POPC. In the asymmetric bilayers the overall percentage of lipids (other than cholesterol) in each leaflet of the bilayer is the same to avoid potential issues with differences in area per lipids. Table S1 shows that the area per lipid after equilibration of the systems. The standard deviation is less than 1% of the average area per lipid for the symmetric systems with similar compositions (PM, PMUpper, PMLower, PM6000). Thus no strain is added to either leaflet of an asymmetric bilayer as a result of non-matching areas per lipid.

### System setup

All simulations were run using the MARTINI CG force field for lipids [60] and proteins [61] and all membranes except one were built by an initial self-assembly of either 1500 or 1759 POPC lipids. For a list and details of simulations see the Supporting Information, Table S1. The 6000 lipid system was created from the 1500 lipid system using the genconf module within gromacs [62] (www.gromacs.org). All systems were solvated by standard MARTINI water beads and neutralized by NaCl to a concentration of 0.15 M. The 1500 lipid plasma membrane simulations (PM, PMUpper, PMLower) consisted of between 35,000 to 47,000 particles, the 6000 lipid plasma membrane simulation (PM6000) consisted of 151,431 particles and the simulations containing proteins were composed of 60,751 particles All simulations except PM6000 were initiated directly from a minimized system. The PM6000 system needed further equilibration to remove steric issues. This was done utilizing built-in free energy functions within gromacs. Initially GM3 and water were removed from the system. The lipids were then gradually equilibrated using the free energy perturbation method, where the presence of all lipids were increased to full van der Waals over 1000 steps using 1 fs. Afterwards the GM3 lipids were included at the positions determined from the exchange lipid protocol (described above) and they were also gradually included using free energy perturbation over 1000 steps using 1 fs time steps. The system was subsequently solvated, neutralized and NaCl was added to a concentration of 0.15 M.

### Simulation details

All simulations were run utilizing gromacs 4.5.x or 4.6.x [62] (www.gromacs.org). The CG MD simulations were performed utilizing the MARTINI version 2.0 lipid CG force field except for PIP_2_, GM3, and with PIP_2_ described in [63]. Parameters for Sph were from the MARTINI force field itp file (http://md.chem.rug.nl/cgmartini/images/parameters/ITP/martini_v2.0_lipids.itp). The ceramide tail from Sph was utilized as the tail for GM3. The head group of GM3 was newly parameterized (see Supporting Information and Fig S7 and S8 for details). Sph and GM3 contains two 4 bead tails with the first bead of the sphingosine tail being unsaturated, while PIP_2_ contains a 4 and a 5 bead unsaturated tail. For an overview of the CG models of all lipids see Fig S9.

In all simulations the pressure was maintained at 1 bar using the Berendsen barostat with a 1 ps coupling constant. In all simulations the temperature was maintained at 310 K and the temperature was controlled by a Berendsen thermostat [64] using a coupling constant of 1 ps. Semi-isotropic pressure coupling was used in all simulations with compressibility of 3×10^−4^ bar^−1^. A time step of 20 fs was used in all the simulation and the van der Waals and coulomb interactions were shifted to zero between 9 Å and 12 Å and 0 and 12 Å respectively. All the simulations were run for 5 µs.

### Simulation analysis

The built-in g_msd function of gromacs was used to calculate the lipid diffusion. The normalized fractional interactions were calculated as the relative number of contacts of a lipid species with each of the other lipid types with a correction for the total number of lipids in the system. Others have previously used this type of calculations to characterize the degree of phase formation in CG simulations [24]. For a two and four component system a fraction of 0.5 and 0.25 respectively correlate to a randomly mixed bilayer. Even though more than one bead was located with the cutoff distance of 11 Å only one contact was registered. To allow for sufficient equilibration only the last 1 µs of the simulation was used from the calculation (4–5 µs) with a 1 ns interval. The nature of such interactions is not necessary symmetric since the density and clustering of specific lipid species will make contacts between lipid A and lipid B different from lipid B interactions with lipid A as a result of number of nearest neighbors and the lipid sizes.

## Cluster distribution

Lipid cluster sizes of GM3 and PIP_2_ within the PM, PMProtein and PM6000 systems during the 5 µs of simulation were calculated using a python implementation of the density based algorithm DBSCAN [65]. Clusters were identified according to the following parameters: the cutoff distance between neighbors was set to 15 Å and the minimum numbers of elements was set to 3. The results was plotted as a function of 4 groups, with 1–3 lipids classified as non-clustered, 4–20 lipids as small clusters, 21–40 as medium clusters and>40 as large clusters (Fig S2 and S3).

The protein cluster sizes were determined during 5 µs using the connectivity networkx python module [66] whereby a point is considered connected to another group of points if within the cutoff distance from at least one the points within the group. The cutoff distance was set to 8 Å for minimum distance between proteins.

## Correlation between bilayer surface curvature and the clustering of lipid molecules

Visualization of the PM6000 simulation suggested a correlation between the curvature in the PM6000 bilayer surface and the clustering of lipid molecules. We quantified this by calculating the cross correlation between the local displacement of the membrane lipids from their average position along the bilayer normal (z) and the local composition of the bilayer within grid boxes evenly distributed across the membrane. The PM6000 lipid was split into 8×8 grid boxes yielding grid boxes of approximately 50 Å^2^. The normalized cross correlation (*R_L,z_*) at a given snapshot was calculated as:
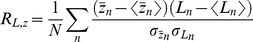
where *L_n_* is the number of lipids of a given species in a grid box, *z_n_* is the z coordinate of the interface between the head groups of the lipids (excluding the current species being calculated) and tails in that box, and the averaging is across all grid boxes. The average *R_L,z_* and the standard deviation over the 5 µs simulation were displayed (Fig. 6) for all lipid species.

Figures were generated in VMD [67].

## Supporting Information

Figure S1 **Fractional interactions of symmetric upper and lower leaflet bilayer simulations.** (A+B) Fractional interactions of the two leaflets within the PMUpper simulation. (C+D) Fractional interactions of lipids within the two leaflets from the PMLower simulation. (E) Outer leaflet of the PMUnsat simulation. (F) Inner leaflet of PMUnsat simulation. (G) Outer leaflet of PM6000 simulation (H) Lower leaflet from the PM6000 simulation (I) Outer leaflet within the PMProtein simulation (J) Lower leaflet within the PMProtein simulation.
(TIF)

Figure S2 **GM3 cluster size.** Cluster size of GM3 using a density based algorithm with the cutoff distance set to 15 Å and the minimum numbers of elements set to 3. Clustering over time is plotted as a function of 4 groups with 1–3 lipids defined as un-clustered, 4–20 lipids as small clusters, 21–40 as medium clusters and>40 as large clusters. The final snapshot with clusters colored according to cluster sizes is shown on the right. (A) PM system (B) PMProtein system (C) PM6000 system.
(TIF)

Figure S3 **PIP_2_ cluster size.** Cluster size of PIP_2_ using a density based algorithm with the cutoff distance set to 15 Å and the minimum numbers of elements set to 3. Clustering over time is plotted as a function of 4 groups with 1–3 lipids defined as being un-clustered, 4–20 lipids as small clusters, 21–40 as medium clusters and>40 as large clusters. The final snapshot with clusters colored according to cluster sizes is shown on the right. (A) PM system (B) PMProtein system (C) PM6000 system.
(TIF)

Figure S4 **Distribution of protein cluster size of protein during 5 µs of simulation.** (A) time evolution of the 16 proteins into clusters colored with respect the cluster size. (B) Size of largest cluster over time.
(TIF)

Figure S5 **Diffusion constant at different time windows of different lipid species within the different simulations.** Standard deviation is included as errorbars. 0–1 µs is shown as squares, 1–2 µs is shown as circles, 2–3 µs is shown as triangle, 3–4 µs is shown upside down triangle, 4–5 µs is shown as rhombus. POPC is shown in dark gray, POPE in red, sphingomyelin in green, GM3 in pink, POPS in blue, PIP_2_ in yellow, DOPC in light gray, DOPE in dark red, DOPS in dark blue. (A) PM, (B) PMUpper, (C) PMLower, (D) PMUnsat, (E) PM6000 and (F) PMProtein.
(TIF)

Figure S6 **Correlation between lipid species within both leaflets of the PM6000 simulation.** Blue indicate that the lipid types within the leaflets are anticorrelated and green indicate there is a correlation between the position of the lipids between the leaflets.
(TIF)

Figure S7 **Structure of GM3 and the bead types used to describe the molecule.** The sugar head group beads are shown in green with the INV particles in gray see Text S1 for details. The ceramide tail is shown in blue.
(TIF)

Figure S8 **Parameter fitting between atomistic and coarse grained simulations of GM3.** (A) Distribution of distances and angles within AT (Top) and CG (bottom) simulations of GM3. The distance and angles are mapped onto the CG structure of GM3 in (B).
(TIF)

Figure S9 **Lipid particle type.** Lipids colored according to their bead types.
(TIF)

Table S1 **Summary of simulations performed.**
(DOCX)

Table S2 **Cholesterol flip-flop.**
(DOCX)

Table S3 **Lipid diffusion coefficients of the six plasma membrane systems.**
(DOCX)

Table S4 **GM3 parameters for gromacs 4.6.**
(DOCX)

Text S1 **Supplementary methodological details.** These provide some details of the parameterization of the coarse-grained model of GM3.
(DOCX)
