# Supplementary figures and images for: Lipid Clustering Correlates with Membrane Curvature as Revealed by Molecular Simulations of Complex Lipid Bilayers
Source: PLoS Comput Biol. 2014 Oct 23;10(10):e1003911. doi: 10.1371/journal.pcbi.1003911 (PMC4207469; doi:10.1371/journal.pcbi.1003911)

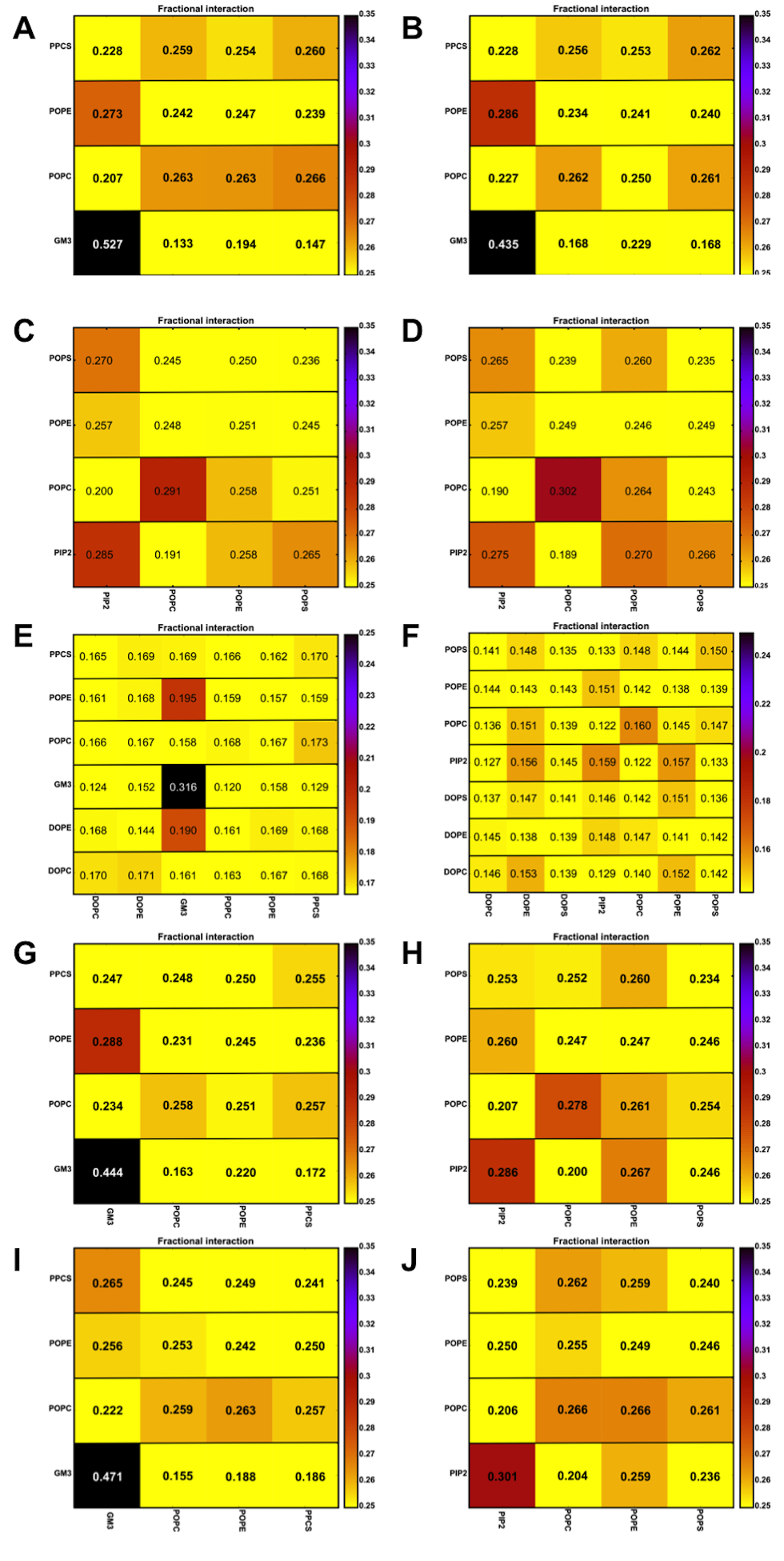

Supplement: Figure S1 — Fractional interactions of symmetric upper and lower leaflet bilayer simulations. (A+B) Fractional interactions of the two leaflets within the PMUpper simulation. (C+D) Fractional interactions of lipids within the two leaflets from the PMLower simulation. (E) Outer leaflet of the PMUnsat simulation. (F) Inner leaflet of PMUnsat simulation. (G) Outer leaflet of PM6000 simulation (H) Lower leaflet from the PM6000 simulation (I) Outer leaflet within the PMProtein simulation (J) Lower leaflet within the PMProtein simulation. (TIF) [file pcbi.1003911.s001.tif]

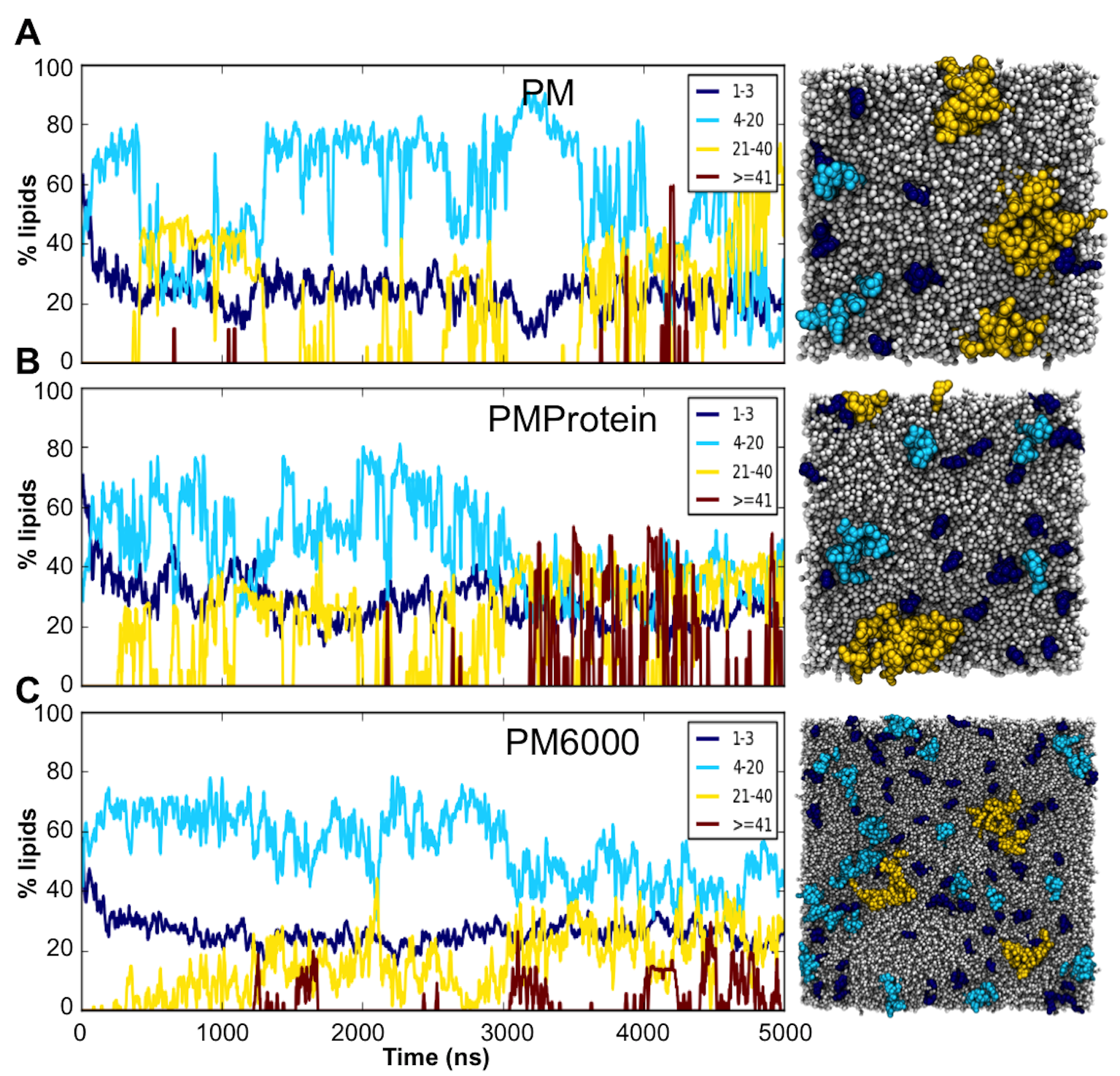

Supplement: Figure S2 — GM3 cluster size. Cluster size of GM3 using a density based algorithm with the cutoff distance set to 15 Å and the minimum numbers of elements set to 3. Clustering over time is plotted as a function of 4 groups with 1–3 lipids defined as un-clustered, 4–20 lipids as small clusters, 21–40 as medium clusters and>40 as large clusters. The final snapshot with clusters colored according to cluster sizes is shown on the right. (A) PM system (B) PMProtein system (C) PM6000 system. (TIF) [file pcbi.1003911.s002.tif]

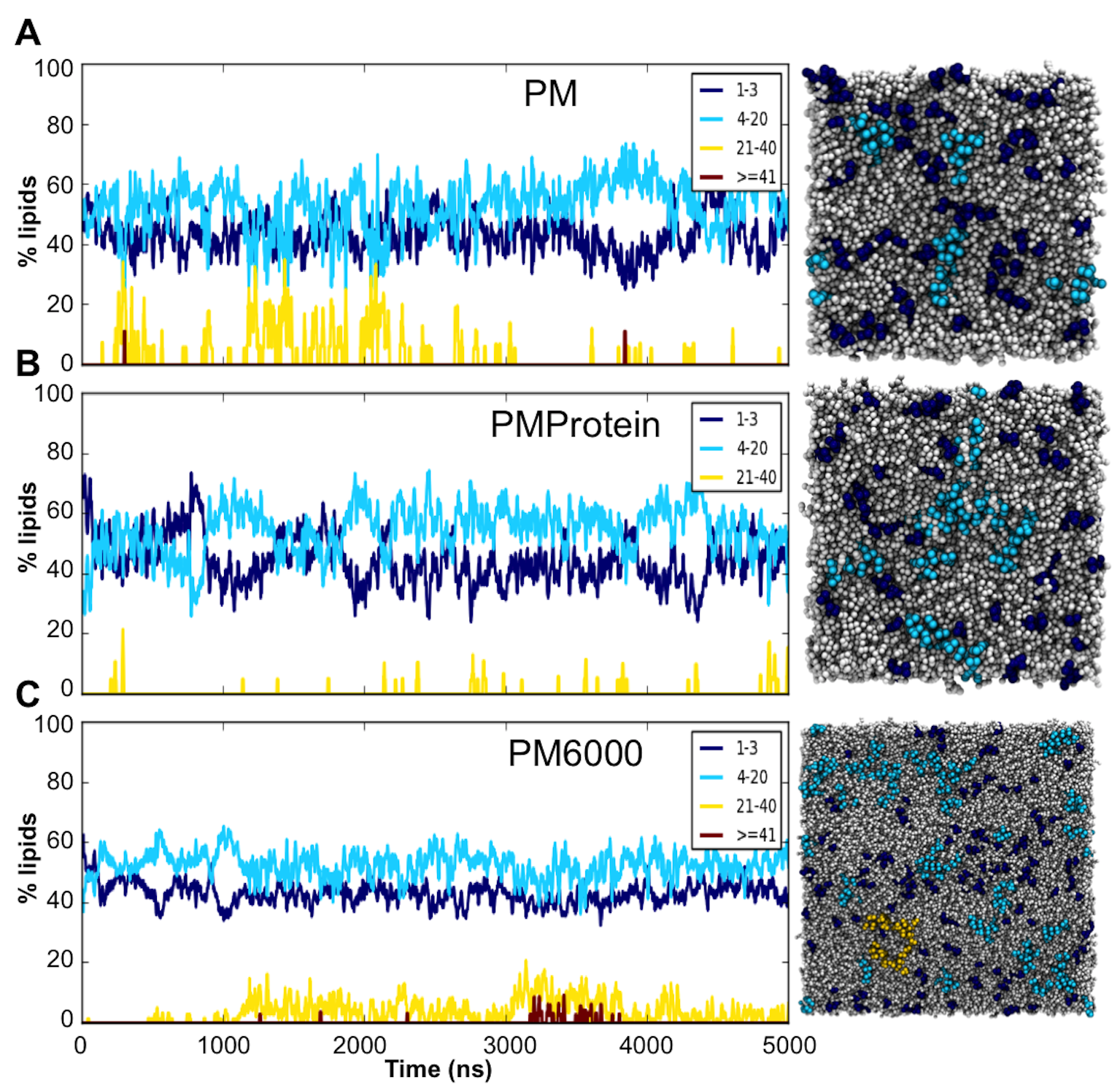

Supplement: Figure S3 — PIP2 cluster size. Cluster size of PIP2 using a density based algorithm with the cutoff distance set to 15 Å and the minimum numbers of elements set to 3. Clustering over time is plotted as a function of 4 groups with 1–3 lipids defined as being un-clustered, 4–20 lipids as small clusters, 21–40 as medium clusters and>40 as large clusters. The final snapshot with clusters colored according to cluster sizes is shown on the right. (A) PM system (B) PMProtein system (C) PM6000 system. (TIF) [file pcbi.1003911.s003.tif]

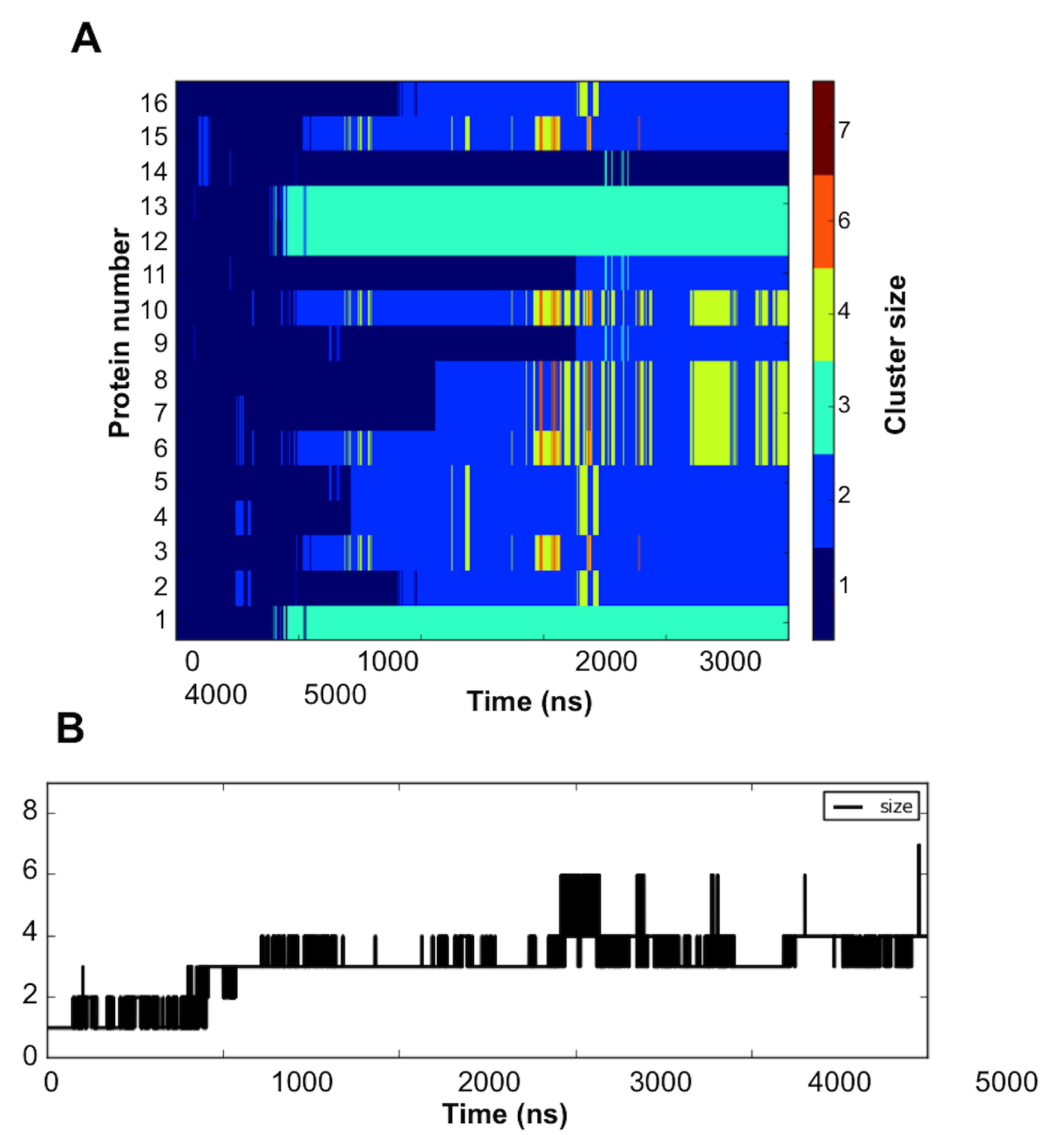

Supplement: Figure S4 — Distribution of protein cluster size of protein during 5 µs of simulation. (A) time evolution of the 16 proteins into clusters colored with respect the cluster size. (B) Size of largest cluster over time. (TIF) [file pcbi.1003911.s004.tif]

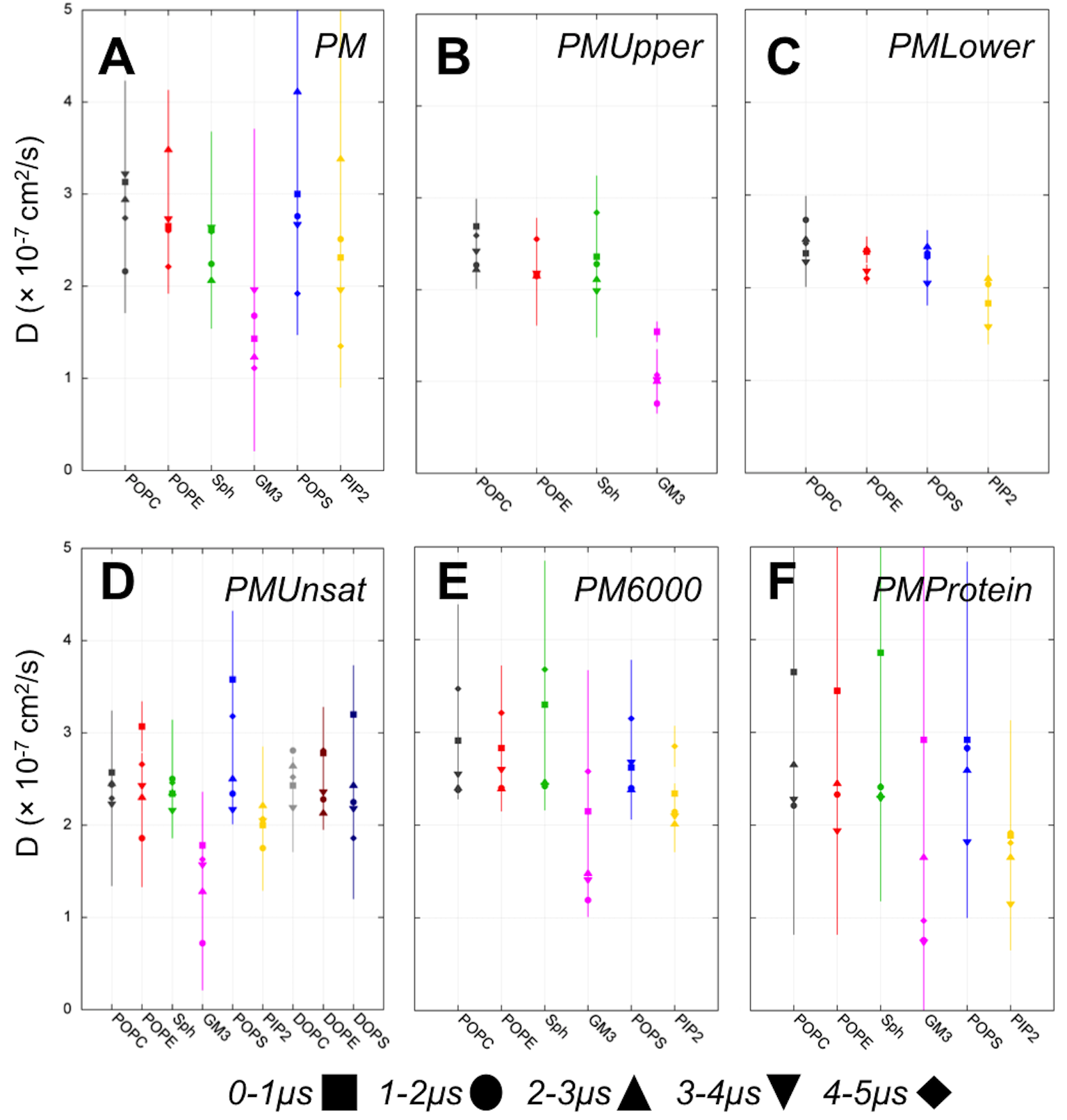

Supplement: Figure S5 — Diffusion constant at different time windows of different lipid species within the different simulations. Standard deviation is included as errorbars. 0–1 µs is shown as squares, 1–2 µs is shown as circles, 2–3 µs is shown as triangle, 3–4 µs is shown upside down triangle, 4–5 µs is shown as rhombus. POPC is shown in dark gray, POPE in red, sphingomyelin in green, GM3 in pink, POPS in blue, PIP2 in yellow, DOPC in light gray, DOPE in dark red, DOPS in dark blue. (A) PM, (B) PMUpper, (C) PMLower, (D) PMUnsat, (E) PM6000 and (F) PMProtein. (TIF) [file pcbi.1003911.s005.tif]

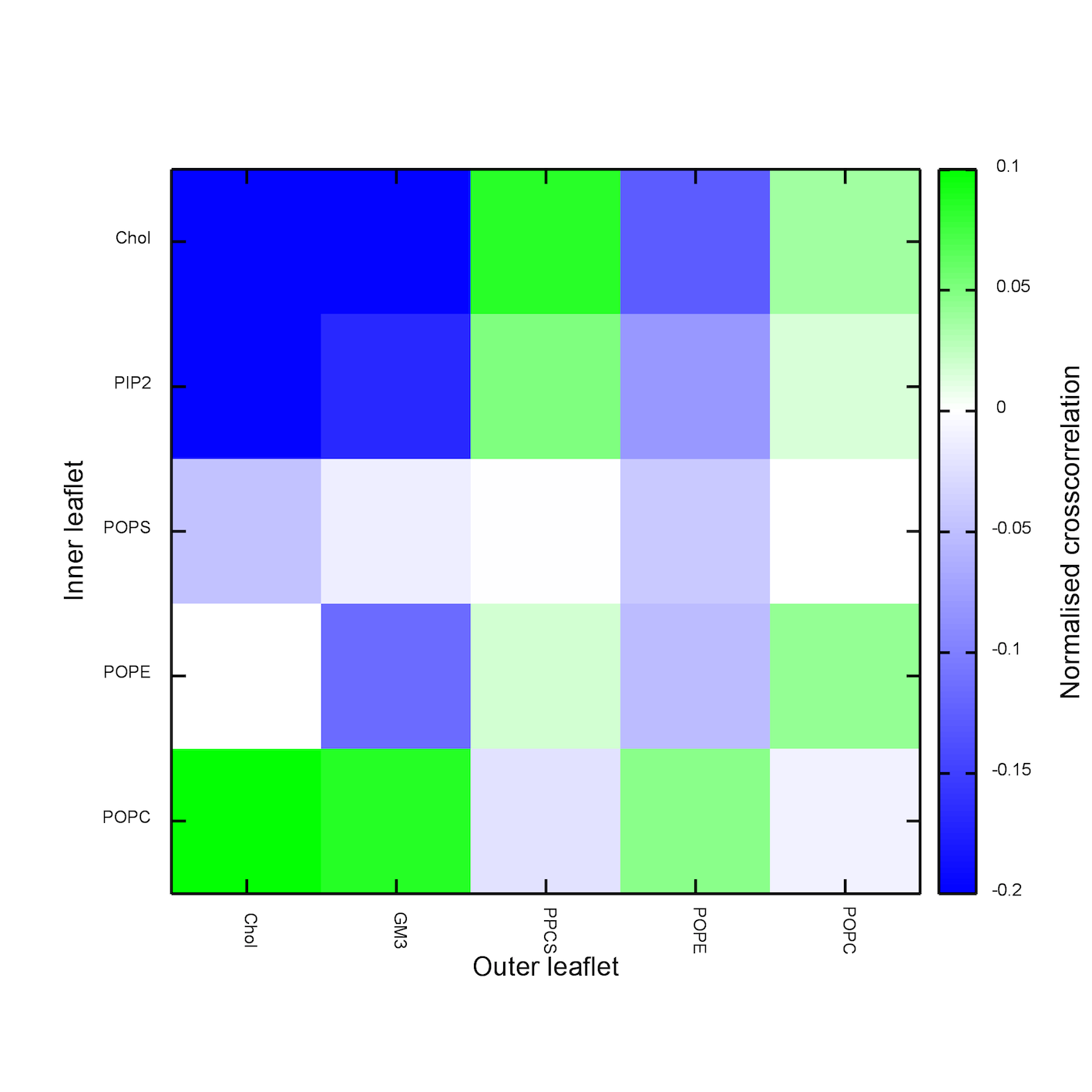

Supplement: Figure S6 — Correlation between lipid species within both leaflets of the PM6000 simulation. Blue indicate that the lipid types within the leaflets are anticorrelated and green indicate there is a correlation between the position of the lipids between the leaflets. (TIF) [file pcbi.1003911.s006.tif]

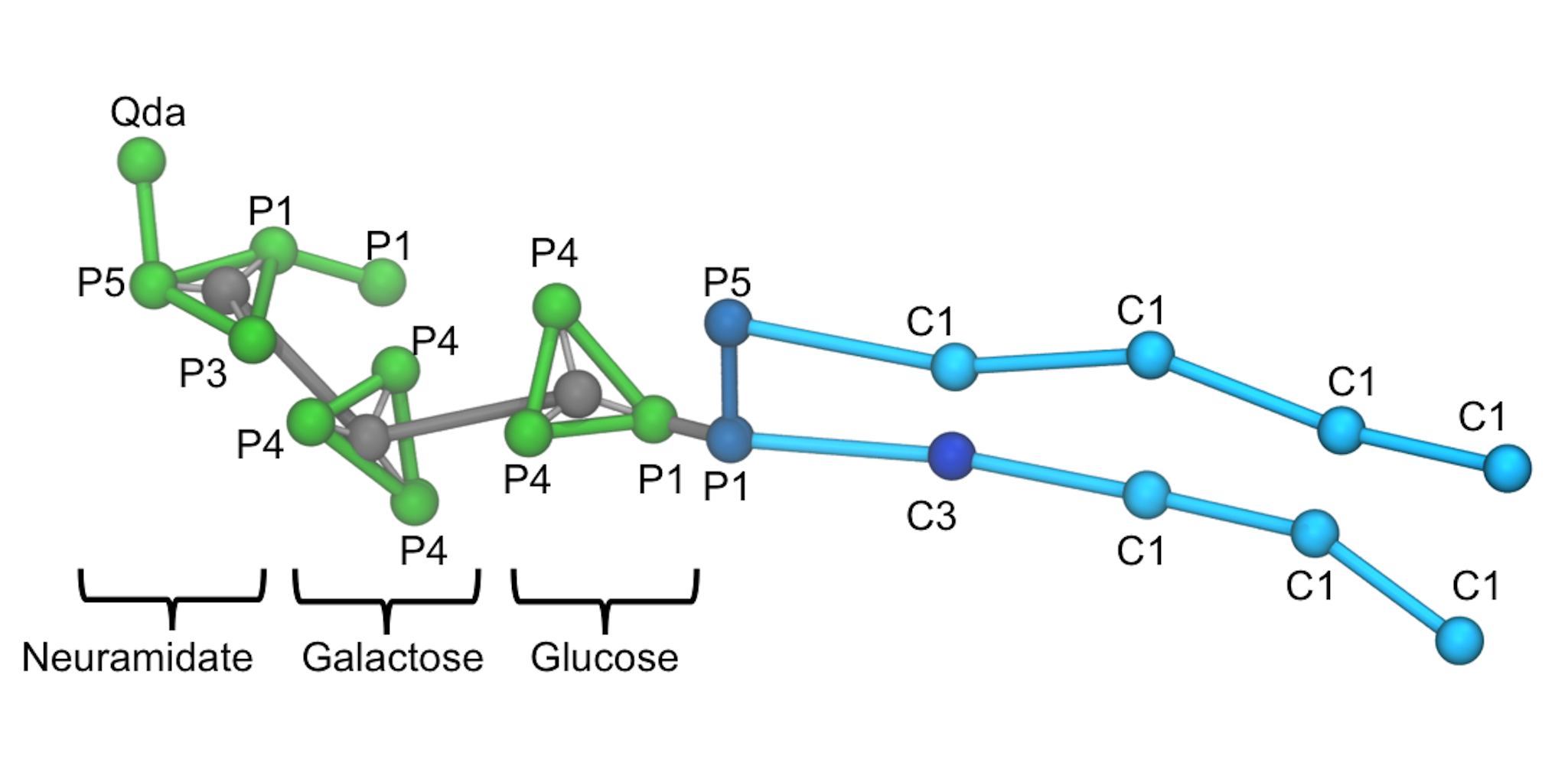

Supplement: Figure S7 — Structure of GM3 and the bead types used to describe the molecule. The sugar head group beads are shown in green with the INV particles in gray see Text S1 for details. The ceramide tail is shown in blue. (TIF) [file pcbi.1003911.s007.tif]

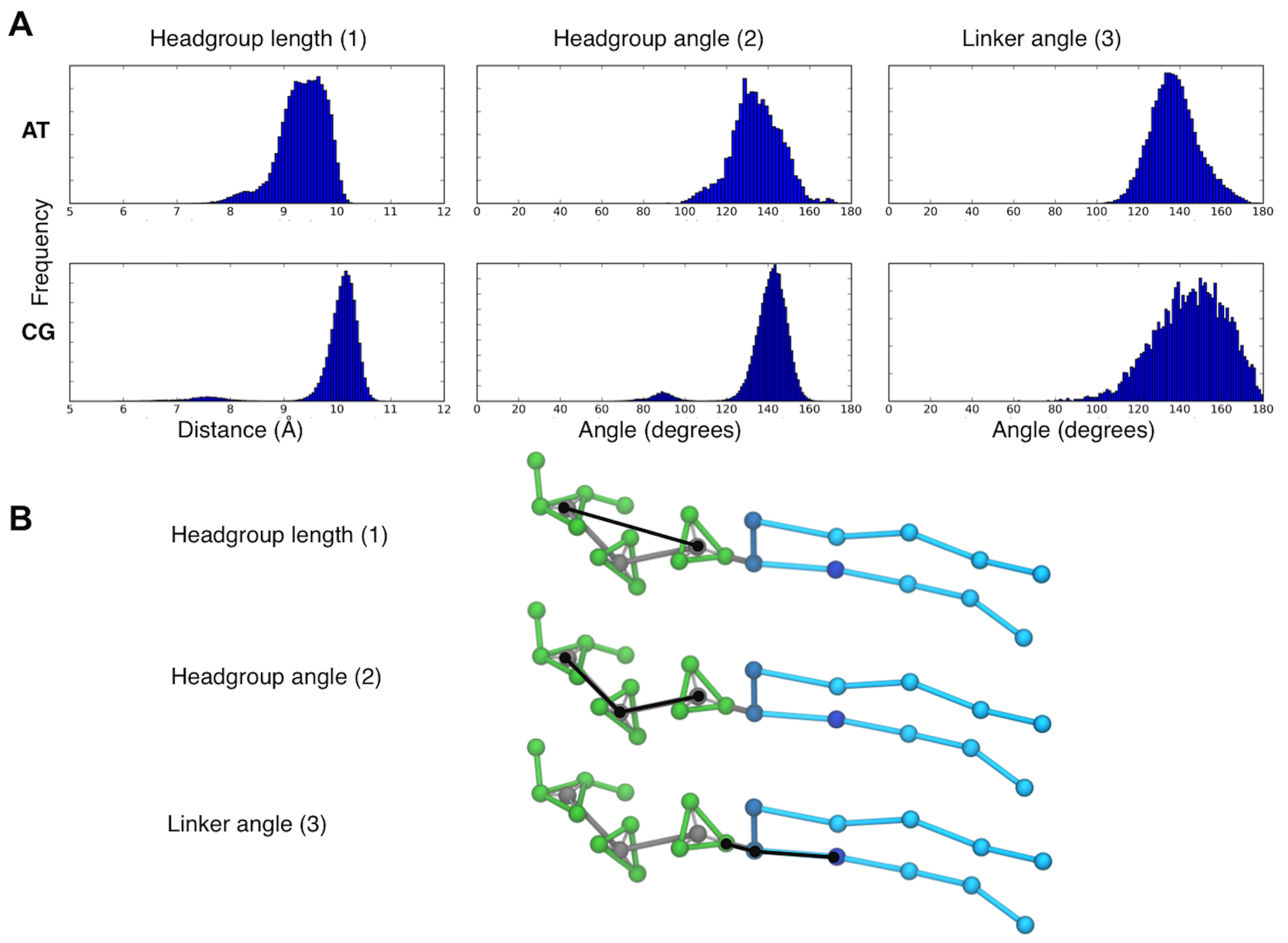

Supplement: Figure S8 — Parameter fitting between atomistic and coarse grained simulations of GM3. (A) Distribution of distances and angles within AT (Top) and CG (bottom) simulations of GM3. The distance and angles are mapped onto the CG structure of GM3 in (B). (TIF) [file pcbi.1003911.s008.tif]

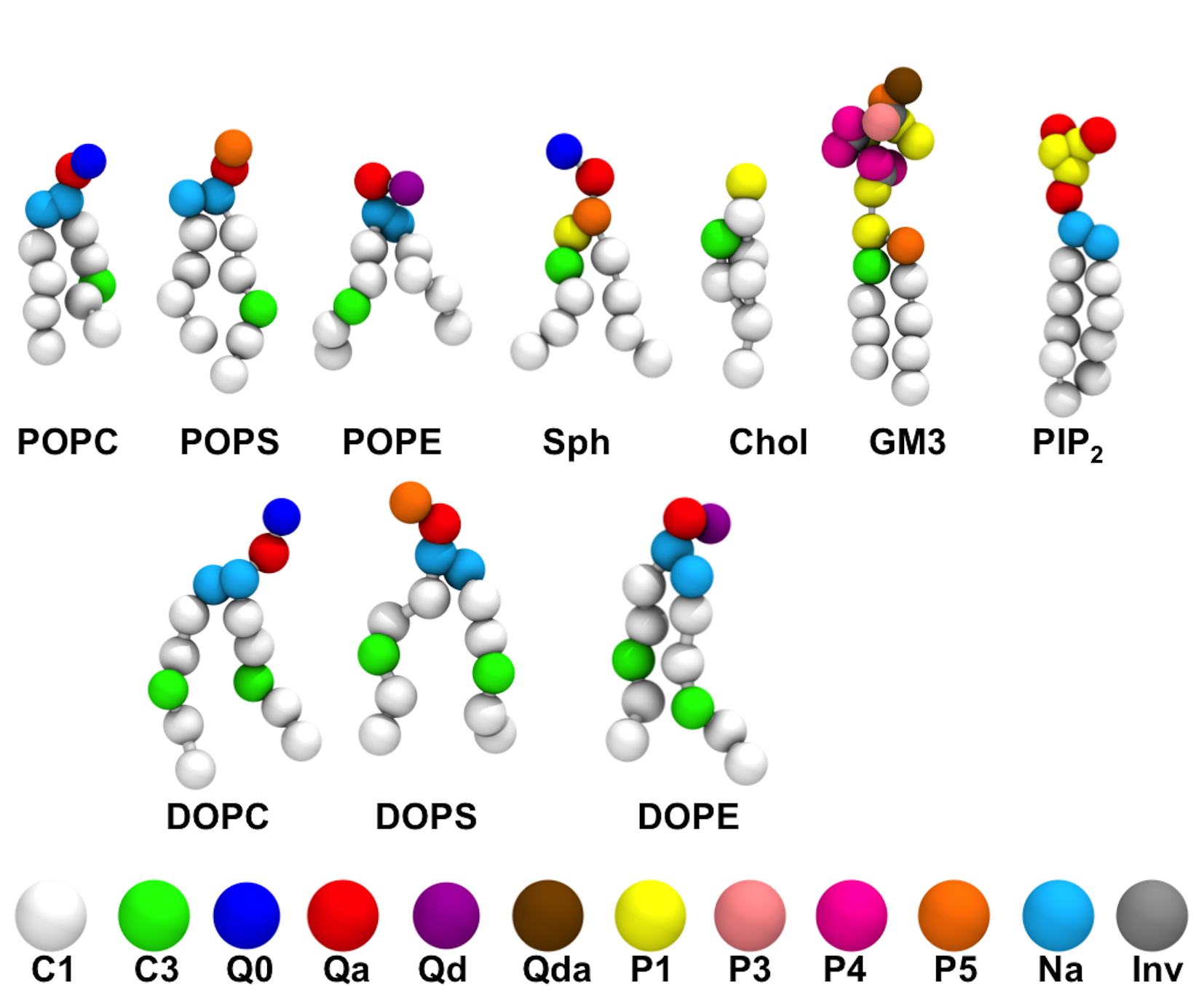

Supplement: Figure S9 — Lipid particle type. Lipids colored according to their bead types. (TIF) [file pcbi.1003911.s009.tif]
