# Supplementary material for: Lipid Clustering Correlates with Membrane Curvature as Revealed by Molecular Simulations of Complex Lipid Bilayers
Source: PLoS Comput Biol. 2014 Oct 23;10(10):e1003911. doi: 10.1371/journal.pcbi.1003911 (PMC4207469; doi:10.1371/journal.pcbi.1003911)
Supplement: Table S1 — Summary of simulations performed. (DOCX) [file pcbi.1003911.s010.docx]

**SI Table S1: Summary of Simulations Performed**

| Simulation | Lipid composition (%) | | | | | | | | | | Total number of lipids | Dimensions  (Å^3^) | Area pr  lipid  (Å^2^) |
| --- | --- | --- | --- | --- | --- | --- | --- | --- | --- | --- | --- | --- | --- |
|  | Outer leaflet | | | | | Inner leaflet | | | | |  |  |  |
| PM | POPC  40 | POPE  10 | Sph  15 | GM3  10 | Chol  25 | POPC  10 | POPE  40 | POPS  15 | PIP2  10 | Chol  25 | 1500 | 193×193×133 | 49.4 |
| PMUpper | POPC  40 | POPE  10 | Sph  15 | GM3  10 | Chol  25 | Same as the outer leaflet | | | | | 1500 | 195×195×130 | 50.6 |
| PMLower | Same as the inner leaflet | | | | | POPC  10 | POPE  40 | POPS  15 | PIP2  10 | Chol  25 | 1500 | 193×193×102 | 49.7 |
| PMUnsat | POPC  20  +  DOPC  20 | POPE  5  +  DOPE  5 | Sph  15 | GM3  10 | Chol  25 | POPC  5  +  DOPC  5 | POPE  20  +  DOPE  20 | POPS  8  +  DOPS  7 | PIP2  10 | Chol  25 | 1500 | 196×196×130 | 51.1 |
| PM6000 | POPC  40 | POPE  10 | Sph  15 | GM3  10 | Chol  25 | POPC  10 | POPE  40 | POPS  15 | PIP2  10 | Chol  25 | 6000 | 386×386×133 | 49.6 |
| PMProtein | POPC  40 | POPE  10 | Sph  15 | GM3  10 | Chol  25 | POPC  10 | POPE  40 | POPS  15 | PIP2  10 | Chol  25 | 1759 | 213×213×173 | N/A |

All simulations were run for 5 µs.
