# Supplementary material for: Lipid Clustering Correlates with Membrane Curvature as Revealed by Molecular Simulations of Complex Lipid Bilayers
Source: PLoS Comput Biol. 2014 Oct 23;10(10):e1003911. doi: 10.1371/journal.pcbi.1003911 (PMC4207469; doi:10.1371/journal.pcbi.1003911)
Supplement: Table S2 — Cholesterol flip-flop. (DOCX) [file pcbi.1003911.s011.docx]

|  | Upper (%) | Lower (%) | Flip-flop rate  (flipflop/ns) | #Chol |
| --- | --- | --- | --- | --- |
| PM | 48.5 | 49.4 | 0.13 | 375 |
| PMUpper | 47.9 | 50.0 | 0.14 | 375 |
| PMLower | 48.1 | 49.6 | 0.11 | 375 |
| PMUnsat | 48.7 | 48.1 | 0.17 | 375 |
| PMProtein | 51.2 | 45.5 | 0.13 | 440 |
| PM6000 | 48.8 | 49.0 | 0.47 | 1500 |

**SI Table S2: Cholesterol flip-flop**
