# Supplementary material for: Lipid Clustering Correlates with Membrane Curvature as Revealed by Molecular Simulations of Complex Lipid Bilayers
Source: PLoS Comput Biol. 2014 Oct 23;10(10):e1003911. doi: 10.1371/journal.pcbi.1003911 (PMC4207469; doi:10.1371/journal.pcbi.1003911)
Supplement: Table S3 — Lipid diffusion coefficients of the six plasma membrane systems. (DOCX) [file pcbi.1003911.s012.docx]

**SI Table S3: Lipid diffusion coefficients of the six plasma membrane systems.**

| Simulation | Diffusion Constant (10^-7^ cm^2^/s) | | | | | | | | |
| --- | --- | --- | --- | --- | --- | --- | --- | --- | --- |
|  | POPC | POPE | PPCS | GM3 | POPS | PIP2 | DOPC | DOPE | DOPS |
| PM | 2.77 (±0.01) | 2.64 (±0.01) | 2.47 (±0.02) | 1.51 (±0.03) | 2.62 (±0.08) | 2.19 (±0.05) |  |  |  |
| PMUpper | 2.45 (±0.03) | 2.32 (±0.03) | 2.43 (±0.01) | 1.35 (±0.01) |  |  |  |  |  |
| PMLower | 2.43 (±0.02) | 2.28 (±0.00) |  |  | 2.24 (±0.00) | 1.85 (±0.01) |  |  |  |
| PMUnsat | 2.50 (±0.01) | 2.50 (±0.02) | 2.49 (±0.03) | 1.39 (±0.06) | 2.70 (±0.01) | 2.07 (±0.01) | 2.61  (±0.05) | 2.47 (±0.01) | 2.45 (±0.01) |
| PM6000 | 2.64 (±0.02) | 2.57 (±0.00) | 2.69 (±0.01) | 1.55 (±0.01) | 2.54 (±0.00) | 2.19 (±0.00) |  |  |  |
| PMProtein | 2.40 (±0.03) | 2.31 (±0.01) | 2.38 (±0.02) | 1.34 (±0.02) | 2.37 (±0.03) | 1.54 (±0.01) |  |  |  |
