# Supplementary material for: Lipid Clustering Correlates with Membrane Curvature as Revealed by Molecular Simulations of Complex Lipid Bilayers
Source: PLoS Comput Biol. 2014 Oct 23;10(10):e1003911. doi: 10.1371/journal.pcbi.1003911 (PMC4207469; doi:10.1371/journal.pcbi.1003911)
Supplement: Table S4 — GM3 parameters for gromacs 4.6. (DOCX) [file pcbi.1003911.s013.docx]

;;;;;;;;;;;;;;;;;;;;;;;;;;;;;;;;;;;;;;;;

;

; GM3 parameters for gromacs 4.6

;

; Please cite:

; H. Koldsø, D. Shorthouse, J. Hélie, M. S. P. Sansom

; ”Lipid Clustering Correlates with Membrane Curvature as Revealed by Molecular

; Simulations of Complex Lipid Bilayers”

; PLoS Comp Biol (2014)

;

;;;;;;;;;;;;;;;;;;;;;;;;;;;;;;;;;;;;;;;;;;;;;;;;;;;;;;;;;;;;;;;;;;;;;;;;;;;;;;;;;;;;;;;;;;;;;;;;;;;;;;;;;

[moleculetype]

;molname exclusions

GM3 1

[ atoms ]

1 P1 1 GM3 B1A 1 0.000

2 P4 1 GM3 B2A 2 0.000

3 P4 1 GM3 B3A 3 0.000

4 Inv 1 GM3 INA 4 0.000

5 P1 1 GM3 B1B 5 0.000

6 P4 1 GM3 B2B 6 0.000

7 P4 1 GM3 B3B 7 0.000

8 Inv 1 GM3 INB 8 0.000

9 P3 1 GM3 B1C 9 0.000

10 P5 1 GM3 B2C 10 0.000

11 P1 1 GM3 B3C 11 0.000

12 Inv 1 GM3 INC 12 0.000

13 P1 1 GM3 B4C 13 0.000

14 Qda 1 GM3 B5C 14 -1.000

15 P5 1 GM3 AM1 15 0.000

16 P1 1 GM3 AM2 16 0.000

17 C1 1 GM3 C1A 17 0.000

18 C1 1 GM3 C2A 18 0.000

19 C1 1 GM3 C3A 19 0.000

20 C1 1 GM3 C4A 20 0.000

21 C3 1 GM3 D1B 21 0.000

22 C1 1 GM3 C2B 22 0.000

23 C1 1 GM3 C3B 23 0.000

24 C1 1 GM3 C4B 24 0.000

[ bonds ]

15 16 1 0.37 1250

15 17 1 0.47 1250

17 18 1 0.47 1250

18 19 1 0.47 1250

19 20 1 0.47 1250

16 21 1 0.47 1250

21 22 1 0.47 1250

22 23 1 0.47 1250

23 24 1 0.47 1250

1 16 1 0.53 3000

1 2 1 0.33 35000

1 3 1 0.37 35000

2 3 1 0.33 50000

5 6 1 0.33 35000

5 7 1 0.37 35000

6 7 1 0.33 50000

9 10 1 0.33 35000

9 11 1 0.37 35000

10 11 1 0.33 50000

10 14 1 0.31 25000

11 13 1 0.29 25000

4 8 1 0.53 30000

8 12 1 0.50 30000

[ constraints ]

1 4 1 0.20

2 4 1 0.16

3 4 1 0.22

5 8 1 0.20

6 8 1 0.16

7 8 1 0.22

9 12 1 0.20

10 12 1 0.16

11 12 1 0.22

[angles]

15 17 18 2 180.0 25.0

17 18 19 2 180.0 25.0

18 19 20 2 180.0 25.0

16 21 22 2 180.0 45.0

21 22 23 2 180.0 25.0

22 23 24 2 180.0 25.0

1 16 15 1 120 45

1 16 21 1 180 45

4 1 16 1 180 45

1 4 8 1 140 200

2 4 8 1 30 200

3 4 8 1 85 200

5 8 4 1 60 200

6 8 4 1 140 200

7 8 4 1 72 200

5 8 12 1 66 200

6 8 12 1 43 200

7 8 12 1 145 200

9 12 8 1 60 200

10 12 8 1 130 200

11 12 8 1 85 200

12 11 13 1 130 250

13 11 9 1 100 250

12 10 14 1 100 250

14 10 9 1 125 250

[ dihedrals ]

4 3 2 1 2 0 1000

8 7 6 5 2 0 1000

12 11 10 9 2 0 1000

1 4 8 5 1 -150 50 1

5 8 12 9 1 -160 50 1
