# Supplementary material for: Lipid Clustering Correlates with Membrane Curvature as Revealed by Molecular Simulations of Complex Lipid Bilayers
Source: PLoS Comput Biol. 2014 Oct 23;10(10):e1003911. doi: 10.1371/journal.pcbi.1003911 (PMC4207469; doi:10.1371/journal.pcbi.1003911)
Supplement: Text S1 — Supplementary methodological details. These provide some details of the parameterization of the coarse-grained model of GM3. (DOCX) [file pcbi.1003911.s014.docx]

**Supplementary Methodological Details:**

*Parameterization of GM3.*

The model for the glycolipid GM3 was produced initially in parts. As GM3 is a glycosphingolipid, the lipid tails and backbone are based on the current model for ceramide tailas found in sphingomyelin which is available from the MARTINI force field developers webpage: (<http://md.chem.rug.nl/cgmartini/images/parameters/ITP/martini_v2.0_lipids.itp>) [[1](#_ENREF_1),[2](#_ENREF_2)] and has further been reported within GM1 [[3](#_ENREF_3)]. The head group consists of the monosaccharides glucose, galactose, and acetylneuraminic acid covalently linked to the ceramide chain. For the CG structure see Fig. S7. Using standard nomenclature, the full structure is:

α-Neu-5-Ac-(2-3)-β-D-Galp-(1-4)-β-D-Glcp-(1-1)-Ceramide

The headgroups were initially constructed based on the monosaccharides parameterised in the sweet martini forcefield[[4](#_ENREF_4)]. Glucose, galactose, and acetylneuraminic acid were all constructed as triangles consisting of polar (P) particles. Glucose and galactose are constructed as a triangle made from two P4 particles and a single P1 particle representing the uneven polarity of the sugar ring. Whilst galactose is not explicitly parameterized previously, the only major difference between it and glucose is the direction in which the C4 OH group faces, having essentially no effect on the CG model. The aNeu5Ac CG model is more complex, due to it not being previously included in sweet martini, and due to the larger side chains present on the model. The ring structure still underlies the CG structure, particle types were chosen based on comparison to known, parameterized, structures. The resultant structure is a ring made up of P3, P5, and P1 particles, with a P1, and Qda particles representing side chains attached to the central ring.

In order to link the carbohydrates together, we opted to model a glycosidic linkage via a system of “rotational nodes”. Thus within each CG triangle we placed a fourth, low mass particle, with no entry on the interactions table – that is, would not have any interaction with any other particle within the system. These “ghost” (INV) particles are anchored within the geometric centre of each ring using constraints, and are then bonded to each other, allowing the rotation and twisting of adjacent sugar molecules through the bond.

Parameters for the bonded and non-bonded interactions of the headgroup were produced by comparison to the atomistic force field GLYCAM [[5](#_ENREF_5)]. By producing a fully atomistic model of the GM3 headgroup with the GLYCAM force field, and simulating it using the AMBER package, we were able to extract details such as bond lengths, angle parameters, and dihedral angles by taking the centre of mass of the constituent particles for each CG bead (Fig S8). As parameters for ceramide tails already exist, the headgroup only, with relevant ceramide backbone was simulated in solvent. Parameters were then fitted to those of the atomistic force field by comparing the two parameter distributions, and adjusting the force constant within the CG force field until the distributions were similar.

The number of angle and dihedral constants was tailored to adjust flexibility of the molecule to roughly physiological levels. Between each pair of sugars, 6 angle parameters were defined (Each one involving both INV particles, and a single ring particle), and a single dihedral angle (ring particle – INV – INV – ring particle) were defined, allowing control over the planar relationship between the two rings in three dimensions.

The linkage between the ceramide backbone and the first carbohydrate (glucose) in the headgroup was also parameterised based on comparison to atomistic simulation. GM3 was simulated in water, for a total of 200 ns, and information about the linkage between the headgroup and tails extracted. From this, angle parameters were applied to the linkage, which was fitted to the atomistic bond distribution (See Figure S8).

This approach was selected to reproduce the behaviour of GM3 since embedding GM3 within a tightly packed bilayer would partially restrain the movement of the sugar moieties, and therefore it would not be as easy to sample enough to get a proper parameter fitting.

A model of GM1 using a different approach was recently published by Marrink and coworkers [[3](#_ENREF_3)]. They however imposed an elastic network to the entire head group to restrain the conformation. Such an extensive restraint was not necessary in this study for GM3 which has a smaller head group than does GM1.

Similar parameterization protocols of other lipid types; including PIP2 [[6](#_ENREF_6)] and cardiolipin [[7](#_ENREF_7)] have previously been shown to reproduce lipid binding sites on proteins that are equivalent to sites validated from crystal structures.

**References**

1. Marrink SJ, Risselada J, Yefimov S, Tieleman DP, de Vries AH (2007) The MARTINI forcefield: coarse grained model for biomolecular simulations. J Phys Chem B 111: 7812-7824.

2. Monticelli L, Kandasamy SK, Periole X, Larson RG, Tieleman DP, et al. (2008) The MARTINI coarse grained force field: extension to proteins. J Chem Theor Comp 4: 819-834.

3. Lopez CA, Sovova Z, van Eerden FJ, de Vries AH, Marrink SJ (2013) Martini force field parameters for glycolipids. J Chem Theor Comput 9: 1694-1708.

4. Lopez CA, Rzepiela AJ, de Vries AH, Dijkhuizen L, Huenenberger PH, et al. (2009) Martini coarse-grained force field: extension to carbohydrates. J Chem Theor Comput 5: 3195-3210.

5. Kirschner KN, Yongye AB, Tschampel SM, Gonzalez-Outeirino J, Daniels CR, et al. (2008) GLYCAM06: A generalizable Biomolecular force field. Carbohydrates. J Comp Chem 29: 622-655.

6. Stansfeld PJ, Hopkinson RJ, Ashcroft FM, Sansom MSP (2009) The PIP_2_ binding site in Kir channels: definition by multi-scale biomolecular simulations. Biochem 48: 10926–10933.

7. Arnarez C, Mazat J-P, Elezgaray J, Marrink S-J, Periole X (2013) Evidence for cardiolipin binding sites on the membrane-exposed surface of the cytochrome bc1. J Amer Chem Soc 135: 3112–3120.
